# ISSPP 2021 2nd Congress of the International Society for the Study of Pleura and Peritoneum

**DOI:** 10.1515/pp-2021-0141

**Published:** 2021-09-27

**Authors:** Janika Kugel

**Rome, Italy, October 7-8, 2021**

**Organizing Committee**

Fondazione Policlinico Universitario A. Gemelli – IRCCS

Division of Peritoneum and Retroperitoneum Surgery – Fabio Pacelli, Andrea Di Giorgio

Division of Gynecologic Oncology – Giovanni Scambia, Giuseppe Vizzielli

* These abstracts have been reproduced directly from the material supplied by the authors, without editorial alteration by the staff of this Journal. Insufficiencies of preparation, grammar, spelling, style, syntax, and usage are the authors.

## BASKET CLINICAL STUDIES AND OTHER HISTOLOGIES

**Abstract BASK-01**

### PREDICTING FACTORS FOR COMPLETION OF PRESSURIZED INTRAPERITONEAL AEROSOL CHEMOTHERAPY (PIPAC) TREATMENT IN PATIENTS WITH PERITONEAL METASTASIS

Authors: Hugo Teixeira-Farinha, Aurélie Balmer, Daniel Clerc, Olivia Sgarbura, Abdelkader Taibi, and Martin Hübner

Institution: Department of Visceral Surgery, Lausanne University Hospital (CHUV), University of Lausanne (UNIL), Switzerland, Surgical Oncology Department, Montpellier Cancer Institute (ICM), University of Montpellier, Montpellier, France, Digestive Surgery Department, Visceral Surgery Department, Dupuytren Limoges University Hospital, Limoges, France

#### Background

The empirical standard for pressurized intraperitoneal (ip) aerosol chemotherapy (PIPAC) consists of three procedures with 4–6 week intervals in between. Completion of treatment has been shown to be prognostic of survival. The aim of this study was therefore to identify predictors for complete treatment at baseline.

#### Methods

Retrospective multicentric cohort study including consecutive patients undergoing PIPAC treatment in Lausanne (Switzerland), Montpellier, and Limoges (France) from January 2015 to January 2020.

#### Results

A total of 183 patients received 517 PIPACs: 30% colorectal, 20% gastric, 10% HPB 32% ovarian, and 8% mesothelioma. Reasons to stop PIPAC treatment were disease progression 48% (16% extraperitoneal), digestive occlusion (14%), patient refusal to continue (11%), other medical reasons (8%), change to cytoreduction and HIPEC 7%, and other (12%). A total of 95 patients (52%) had ≥3 PIPACs (pp: per protocol) and were compared to 88 patients with 1 or 2 treatments. Median overall survival (OS) was better in per protocol (15 vs 7 months, p=0.001). On univariate analysis, pp patients had less ascites (mean: 380ml±100 vs 960ml±188; p=0.001) and less history of ileus (12 vs 24%; p=0.01) Significant predictors become non-significant in multiple regression.

#### Conclusion

Stopping PIPAC associates with worse prognosis and reasons for stopping treatment are multifold. Elaboration of a prediction score for complete PIPAC treatment is part of the ongoing international PIPAC cohort study.

**Abstract BASK-02**

## PLUS STUDY: PRELIMINARY RESULTS ABOUT FEASIBILITY AND SAFETY OF CYTOREDUCTIVE SURGERY AND PIPAC

Authors: - Manuela Robella1, Martin Hubner2, Marc Reymond 3, Andrea di Giorgio 4, Olivia Sgarbura 5, Vladimir Khomiakov 6, Aditi Bhatt 7, Wouter Willaert 8, Naoual Bakrin 9, Mohammad Alyami 9, 10, Michele De Simone Michele 1, and Marco Vaira 1.

Institution: 1 Unit of Surgical Oncology, Candiolo Cancer Institute, FPO – IRCCS. 2 Department of Visceral Surgery, Lausanne University Hospital, University of Lausanne, Switzerland, 3 Department of General & Transplant Surgery, University Hospital Tübingen, Germany, 4 Surgical Unit of Peritoneum and Retroperitoneum, Fondazione Policlinico Universitario A. Gemelli IRCCS, Rome, Italy, 5 Department of Surgical Oncology, Cancer Institute Montpellier (ICM), IRCM, Institut de Recherche en Cancérologie de Montpellier, INSERM U1194, Université de Montpellier and Institut régional du Cancer de Montpellier, France, 6 Department of thoracoabdominal cancer surgery, P.A. Hertsen Moscow Research Oncological Institute – branch of the National Medical Research Center of Radiology, Moscow, Russia, 7 Department of Surgical Oncology, Zydus Hospital, Ahmedabad, India, 8 Department of GI Surgery, Ghent University Hospital, Ghent, Belgium, 9 Department of General Surgery and Surgical Oncology, Center Hospitalier Lyon- Sud, Hospices Civils de Lyon, Pierre-Bénite, France,10 Department of General Surgery and Surgical Oncology, Oncology Center, King Khalid Hospital, Najran, Saudi Arabia.

### Background

PIPAC is a minimally invasive approach relying on physical principles for improving ip drug delivery. Feasibility and safety are now consolidated and data on its effectiveness are continuously increasing. Although any surgical procedure associated with PIPAC had always been discouraged due to the high risk of complications, with the growth of expertise, more and more surgical teams associate PIPAC with surgery.

### Methods

The Plasma-Lyte 148® versUs Saline (PLUS) study has collected the experience of 10 centers around the word (Italy, Switzerland, Germany, Russia, France, Belgium, Saudi Arabia, and India) and 96 cases of PIPAC + cytoreductive surgery (CRS) were included. The procedures most frequently performed were adhesiolysis (n=41), adnexectomy (n=29), umbilical/inguinal hernia repair (n=14), intestinal resections/repairs (n=7), gastrectomy (n=7), and splenectomy(n=1).

### Results

PLUS study demonstrated, through a propensity score analysis, that PIPAC associated to cytoreductive surgery (CRS) is linked to an increase of surgical time, length of stay and medical complication rate; the most frequently reported medical complications were mild or moderate in severity, such as abdominal pain, nausea, ileus, and hyperthermia. No difference in terms of surgical complications was registered; neither reoperations nor postoperative deaths were reported.

### Conclusion

If these evidences are confirmed, new scenarios for the application of PIPAC combined with surgical cytoreduction will open up.

**Abstract BASK-03**

## WHY AND WHEN COULD PATIENTS WITH PERITONEAL METASTASES FROM BREAST CANCER BENEFIT FROM CYTOREDUCTIVE SURGERY? A MULTICENTER SURVEY

Authors: Maurizio Cardi, Marc Pocard, Rea Lo Dico, Andrea Di Giorgio, and Paolo Sammartino.

Institution: Dipartimento di Chirurgia P. Valdoni. Sapienza Università di Roma, University of Paris,

UMR 1275 CAP Paris Tech Metastasis Peritoneum Paris Technology, Unità Chirurgia Peritoneo e Retroperitoneo. IRCCS Fondazione Policlinico Universitario A. Gemelli, Roma

### Background

Even though breast cancer (BC) is the most frequent extraabdominal tumor causing peritoneal metastases, clear clinical guidelines are lacking. Our aim is to establish whether CRS could be considered as a treatment option in selected patients with peritoneal metastases from breast cancer (PMBC).

### Methods

We considered patients with PMBC treated in 10 centers from January 2002 to May 2019. Clinical data included BC characteristics (age, histology, and TNM), and on metastatic disease (interval BC-PM, molecular subtype, other metastases, peritoneal spread). OS was estimated using the Kaplan–Meier method. Univariate and multivariable data for OS were analyzed.

### Results

Of the 49 women with PMBC, 20 treated with cytoreductive surgery and 29 with noncurative procedures, the 10-year OS rate was 27%. Patients treated with curative intent had a better OS than patients treated with noncurative procedures (89.2% vs. 6% at 36 months, p <0.001). Risk factors significantly influencing survival were age at primary BC, interval between BC and PM diagnosis, extraperitoneal metastases, and molecular subtype.

### Conclusion

The improved outcome in selected cases after a multidisciplinary approach including surgery should lead researchers to regard PMBC patients with greater attention. Our collective efforts give new information, suggest room for improvement and point to further research for a hitherto poorly studied aspect of metastatic BC.

**Abstract BASK-03**

## CONSENSUS STATEMENT ON SAFETY MEASURES FOR PRESSURIZED INTRAPERITONEAL AEROSOL CHEMOTHERAPY

Authors: A. Girardot-Miglierina*, D. Clerc*, M. Alyami, L. Villeneuve, O. Sgarbura, M.A. Reymond, and M. Hübner

*Equal contribution

Institution: Department of Visceral Surgery, Lausanne University Hospital CHUV, University of Lausanne (UNIL), Switzerland

### Background

PIPAC is a promising treatment for peritoneal cancer that entails, however, potential risks for the caregivers in the operating room (OR). This study aimed to reach an expert consensus on a comprehensive safety protocol.

### Methods

Active PIPAC centers were invited to participate in a two-round Delphi process on 43 predefined items: summary of the existing evidence was presented along with questions according to the PICO framework. Strength of recommendation was determined through a 2-round Delphi process, accepting a consensus threshold of ≥50% of agreement for any of the four options (GRADE), or ≥70% in either direction.

### Results

Forty-seven out of 66 invited panelists (76%) answered both rounds. Consensus was reached for 41/43 items (95.3%). Strong and weak recommendations were issued for 30 and 10 items, respectively. One recommendation reached consensus with combined agreement. No consensus was reached for 2 items. Strong positive recommendations were issued for all items related to information and training requirements on PIPAC safety aspects, and for all involved caregivers.

### Conclusion

High degree of consensus was reached for a comprehensive PIPAC safety protocol, adapted to the risk of exposure for the different caregivers in the OR. This consensus can serve as a basis for education and help to reach a high degree of adherence in daily practice.

**Abstract BASK-04**

## PRESSURIZED INTRAPERITONEAL AEROSOL CHEMOTHERAPY (PIPAC): SURGICAL SECURITY FLOW-CHART AND LITERATURE SURVEY

Authors: Delgadillo Xavier^1^ Gaspar Tunde^1^ Wüthrich Philippe^2^

Institution: Center Médico Chirurgical Volta, La Chaux de Fonds, Switzerland. ^2^ Center Hospitalier Genolier, 1273- Arzier, Switzerland.

### Background

PIPAC is a very well-tolerated procedure. Important Surgical Security flow charts are needed to present as an emergency guide:

### Methods

We had determined to follow both “how-to” guidelines as well as algorithm-based guidelines to understand the thought process behind the proposed treatment strategy. How to avoid being in tough surgical situations through preoperative planning, how to better deal with commonly encountered intraoperative findings, how to deal with difficult laparoscopic, open and surgical cases, and to avoid medico-legal issues concerns directly our group.

### Results

We bring simple and succinct pragmatic and reproducible techniques that can be readily implemented by all surgeons of varying experience to successfully treat complex situations that may make the difference in patient outcomes in case of PIPAC procedure, after an exhaustive literature survey. Before start describing the target & the flowchart’s main objective, we consider to awake all conditions needed. It is important to prepare yourself for the “first cases”. Training workshops and instruction are fundamental and mandatory to obtaining the certificate to perform a PIPAC.

### Conclusion

It is very useful to have a flow chart algorithm to follow step by step a PIPAC procedure, without any adverse event, in the case of arrival of any special situation; we consider that this initial flow chart is very useful in case of the beginning of PIPAC experience.

**Abstract BASK-05**

## RESULTS OF SIMULTANEOUS ADRENAL RESECTION IN CYTOREDUCTIVE SURGERY AND HIPEC PATIENTS DUE TO PERITONEAL METASTASIS

Authors: Özgül Düzgün1, Pırıltı Özcan2, Murat Kalın2, and Ömer Faruk Özkan 2.

Institution: 1 Health Science University, Ümraniye Training and Research Hospital, Surgical Oncology, İstanbul, Turkey.

2 Health Science University, Ümraniye Training and Research Hospital, General Surgery, İstanbul, Turkey.

### Background

Our aim is to present the results of patients with peritoneal metastasis who had CRS and hyperthermic intraperitoneal chemothreapy (HIPEC) due to abdominal tumors without distant metastasis.

### Methods

In between April 2016-2021 in Umraniye Research and Training Hospital, simultaneous adrenalectomy was performed to 11 cases among 287 cases with CRS+HIPEC operation due to different etiological causes. These 11 cases were evaluated on the basis of age, gender, etiology, operation time, amount of bleeding, and chemotherapy protocol applied during HIPEC, simultaneous adrenalectomy methods, mortality, and morbidity.

### Results

Among 11 cases, 7 of them were women, 4 of them were men. Age average was 55 (age interval between 23–71). Primary diagnoses were as follows: 4 of these cases had ovarian cancer, 3 of them had colorectal cancer, 2 of the cases had gastric cancer, 1 of the cases had mesothelioma, and 1 of the cases had sarcomatosis. Average operation time was 6.9 hours (time interval differed from 4-12 hours). Average bleeding amount was 550 cc(ranged between 250–2000). Among the patients who had simultaneous adrenalectomy during CRS +HIPEC, 8 of the cases had right adrenalectomy and 3 of them had left adrenalectomy. None of the patients had bilateral adrenalectomy. İntravenous and ip FOLFOX protocol was applied to patients with colorectal cancer and ip cisplatin and doxorubicin(DXR) were applied to other – TRUNCATED 1,500 CHARACTERS

**Abstract BASK-06**

## OUTCOME OF PATIENTS (PTS) WITH UNCOMMON HISTOLOGIES (UH) TREATED WITH CYTOREDUCTIVE SURGERY (CRS) AND HEATED INTRAPERITONEAL CHEMOTHERAPY (HIPEC)

Authors: Piero Vincenzo Lippolis 1, Silvia Cesario 2, Pinuccia Faviana 3, Laura Boldrini 3, and Francesco

Forfori 4, Augusto Brogi 4, Virginia Genovesi 2, Valentina Massa 2, Francesca Salani 2, and L. Piccini 1,

E. Rreka 1, Lorenzo Fornaro 2, and Gianluca Masi 2

Institution: 1 SC Multidisciplinary Clinical Center for Peritoneum Surgery - SD General and

Peritoneal Surgery, AOUP, 2 U.O. Medical Oncology, AOUP, 3 U.O. Pathological Anatomy III Univ.,

AOUP, 4 U.O. Interdepartmental Anesthesia and Resuscitation Univ., Azienda Ospedaliero-Universitaria Pisana, Pisa, Toscana, Italy

### Background

Peritoneal metastasis (PM) is associated with poor prognosis in several cancers. The safety and efficacy of CRS/HIPEC have not been evaluated in pts with uncommon histologies (UH).

### Methods

We reviewed all pts with PM from UH treated with CRS/HIPEC at our center.

### Results

From March 2016 to April 2021 170 CRS/HIPEC procedures were performed. Seven pts with UH were identified: Cholangiocarcinoma (1), hepatocellular carcinoma (3), adrenal pheocromocytoma (1), clear cell sarcoma (1), and liposarcoma (1). Median age was 56 years. PM was isolated (4 pts) or with liver metastases (3 pts). Median PM index was 9. All pts received available standard treatments (ST), with median disease control of 17.7 months. Eligibility for CRS/HIPEC was assessed by multidisciplinary team. All pts received DXR, while cisplatin was used for cholangiocarcinoma. Liver lesions were resected or ablated intraoperatively. Macroscopically complete resection was achieved in all pts. Clavien–Dindo grade 3 complications occurred in 2 pts (wound dehiscence). After a median follow-up of 15.5 months, 5 pts were alive with no peritoneal recurrence (PR). Two pts developed PR after 1.5 and 2.1 months and died due to progression 17.3 and 11.2 months after CRS/HIPEC.

### Conclusion

CRS/HIPEC is feasible in selected pts with UH. Tumor biology and disease control with ST should guide multidisciplinary case-by-case discussion. Dedicated studies will define the role of CRS/HIPEC in this setting.

## GASTRIC CANCER PERITONEAL METASTASES

**Invited lecture**

**CONTROVERSIES OF HIPEC FOR GASTRIC CANCER PERITONEAL METASTASIS**

Speaker: Beate Rau MD, PhD

Institution: Department of Surgery, Campus Virchow-Klinikum and Charité Campus Mitte, Charité -Universitätm2edizin Berlin, Berlin, Germany

### Content

Patients with peritoneal metastases of gastric cancer have a poor prognosis and a median survival of about 7 months. CRS in combination with HIPEC showed promising results in selected patients. However, HIPEC has not shown significant effect in randomized trials when Oxaliplatin was used for 30 minutes. Therefore, HIPEC as an additional tool to improve survival in patients with gastric cancer and peritoneal metastasis is still on discussion. We will discuss recent results and point out the limitation of the recent literature.

**Invited lecture**

## NEOADJUVANT LAPAROSCOPIC HIPEC FOR GASTRIC CANCER PERITONEAL METASTASES

Speaker: Brian Badgwell, MD, MS

Institution: MD Anderson Cancer Center, Houston, TX. USA

As the peritoneum is the most common site of metastatic disease for gastric cancer, ip therapy has been an area of interest for many investigators. There are theoretical benefits to a neoadjuvant approach, such as identifying patients most suitable for aggressive surgery and frequent use of diagnostic laparoscopy in evaluating peritoneal disease. Therefore, the purpose of our peritoneal program was to investigate the use of laparoscopic HIPEC in a neoadjuvant approach prior to consideration of gastrectomy. In our first phase II trial (NCT02092298), we repeated laparoscopic HIPEC until peritoneal disease was undetectable, and then performed gastrectomy alone (without concomitant HIPEC). Out of 19 patients, 5 underwent gastrectomy, with a median survival of 30 & 20 months from date of metastatic diagnosis and first laparoscopic HIPEC, respectively. Our second phase II trial (NCT02891447) was a more aggressive approach of offering cytoreduction, gastrectomy, and HIPEC after at least one laparoscopic HIPEC. Out of 20 patients, we reported a 3-year survival rate of 28%. Lastly, we recently completed a phase I trial of laparoscopic HIPEC with triplet chemotherapy (NCT03330028) to incorporate paclitaxel into our standard cisplatin and mitomycin regimen. In summary, although recently completed early phase trials are promising, cooperative group and multi institutional registry efforts are needed to help address this clear area of unmet need.

**Invited lecture**

## THE ROLE OF ONCOLYTIC VIRUSES IN INTRAPERITONEAL THERAPY

Authors: Yanghee Woo, MD; Shyambabu Chaurasiya, PhD; Zhifang Zhang, PhD; Annie Yang, MD; and Yuman Fong

Institution: City of Hope, Duarte, CA, USA

Oncolytic viral therapy is promising field of anticancer immunotherapy. Immuno-oncolytic viruses (IOVs) possess inherent selective cancer killing and the genetically modified capacity to deliver desired human transgenes such as hNIS and antiPDL1. This versatility allows for tumor tracking and enhancing antitumor immunity. We have created a novel IOV platform, CF33 and investigated its potential to treat GI PM. CF33 is an artificially created replication-competent chimeric orthopoxvirus with a large DNA genome. Genetically modified variants, CF33-hNIS and CF33-hNIS-antiPDL1 exhibited a large therapeutic window starting at doses orders of magnitudes lower than those required by other oncolytic viruses (OVs) and the capacity to decrease tumor burden and improve survival. In an aggressively fatal PDAC PM nude mouse model with a subcutaneous (SQ) and peritoneal tumors, various routes (IV, IP, intrathecal [IT]) of CF33-hNIS-antiPDL1 administration resulted in the most robust peritoneal tumor kill with IT injection of SQ tumor (data not published) and least effectiveness with IV. We confirmed that CF33-hNIS-antiPDL1 can not only track and kill peritoneal tumors, but express functional hNIS for imaging of PM and locally deliver antiPDL1. Finally, studies of tumor immunogenicity have revealed that CF33-IOVs alter the immune tumor microenvironment including upregulation of PD-L1 for potential synergistic antitumor immunity. In summary, CF33-IOV-platform demonstrates promising preclinical results against PM.

**Abstract GC-01**

## RESULTS OF PRESSURIZED INTRAPERITONEAL AEROSOL CHEMOTHERAPY (PIPAC) FOR GASTRIC CANCER WITH PERITONEAL METASTASES

Authors: Jarrod KH Tan, Guowei Kim, Xin-Yi Chan, Aileen Pang, Raghav Sundar, Hon Lyn Tan, Wei, Peng Yong, and Jimmy BY So

Institution: National University Health System, Singapore

### Background

PIPAC is an innovative ip chemotherapy delivery technique. Intravenous nivolumab is a treatment modality for progressive gastric cancer after conventional chemotherapy. PIPAC-oxaliplatin (OXA) in combination with nivolumab as an immune checkpoint inhibitor may improve immune activation in gastric cancer peritoneal metastasis (GCPM) patients.

### Methods

Prospective phase I trial of GCPM patients after failure of at least first-line chemotherapy. Patients were treated with either PIPAC-OXA (Cohort-1) or in combination with nivolumab (Cohort-2). Safety and clinicopathological response was analyzed.

### Results

A total of 14 patients were recruited, 8 and 6 in cohort-1 and cohort-2, respectively. Median Peritoneal Cancer Index (PCI) score was 15. In Cohort-1, there were no dose limiting toxicities and the highest dose group (120mg/m2) tolerated PIPAC well. Three patients underwent a second PIPAC. One patient had marked improvement in PCI from 30 to 12, while the remaining two patients had stable PCI/peritoneal regression grading (PRGS) scores. On the basis of response evaluation criteria in solid tumor (RECIST), 62.5% and 66.7% had stable disease after one and two PIPAC procedures respectively. In cohort-2, patients underwent combination therapy with oxaliplatin 90mg/m2. No serious adverse events were recorded. The results of the treatment response are currently being evaluated.

### Conclusion

PIPAC-OXA in combination with IV nivolumab can be safely administered to GCPM patients. Further analyses are required to delineate its efficacy in GCPM.

**Abstract GC-02**

## PRESSURIZED INTRAPERITONEAL AEROSOL CHEMOTHERAPY (PIPAC) FOR UNRESECTABLE PM FROM GASTRIC CANCER, OUTSTANDING RESULT FROM MULTICENTER COHORT STUDY

Authors: Mohammad Alyami 1, Vladimir Khomiakov 2, Alexandru Lintis 3, Pompiliu Piso 4, Clarisse Eveno 3, Olivier Glehen 5, and the ISSPP PIPAC cohort study group

Institutions: 1. Department of surgery and Surgical Oncology, King Khalid hospital, Najran, Saudi Arabia 2. Department of thoracoabdominal cancer surgery, P.A. Hertsen Moscow Oncology Research Center, Moscow, Russia. 3. Department of Digestive and Oncological Surgery, Claude Huriez University Hospital, Lille, France. 4. Department of Surgery, Krankenhaus Barmherzige Brüder Regensburg, Regensburg, Germany. 5. Department of General Surgery and Surgical Oncology, Center Hospitalier Lyon-Sud, Hospices Civils de Lyon, Pierre-Bénite, France.

### Background

PIPAC has promising results for patients with PM. It is safe and well tolerated. Our aim was to report oncological outcomes after PIPAC for gastric PM.

### Methods

International retrospective cohort study of patients with gastric PM. Outcome measures were OS, radiological response (RECIST), histological response by PRGS and cytology.

### Result

A total of 586 nonselected patients with a median age of 56 years, 54% of them were female and underwent a total of 1566 PIPAC procedures. A total of 37% of patients were treated with ≥2 lines of IV chemotherapy, median PCI at first PIPAC was 14 (7-24), and 63% of them were with signet ring adenocarcinoma. Grade III-IV morbidity was 5.1 % and 1.9% died within 30 days from PIPAC procedure. Median OS was 15.4 months from diagnosis and 20.1 months for patient with >3 PIPAC. 263/586 patients (44.9%) had ≥3 procedures (pp: per protocol) with the following outcomes: RECIST: 4.3% complete response 11% partial remission, 44% stable; PRGS1 39% at PIPAC3 and negative cytology at PIPAC3 in 16% of patient. In multivariate analysis, 3 PIPAC or more HR 0.3 (95% CI 0.27–0.51), 2nd and 3rd line of chemotherapy HR 0.48 (95% CI 0.25–0.92) and CRS&HIPEC after PIPAC HR 0.3 (95% CI 0.18-0.51) were predictors for survival.

### Conclusion

Based on this large multicenter cohort study, PIPAC could be considered as an option for the treatment of PM from gastric cancer. More prospective studies are needed to validate this indication.

**Abstract GC-03**

## OUTCOMES OF A PHASE II STUDY OF INTRAPERITONEAL PACLITAXEL PLUS SYSTEMIC CAPECITABINE AND OXALIPLATIN (XELOX) FOR GASTRIC CANCER WITH PERITONEAL METASTASES

Authors: Daryl KA Chia, Raghav Sundar, Guowei Kim, Jia Jun Ang, Jeffrey HY Lum, Min En Nga, Giap Hean Goh, Seet Ju Ee, Cheng Ean Chee, Hon Lyn Tan, Jingshan Ho, Natalie YL Ngoi, Matilda, XW Lee, Vaishnavi Muthu, Gloria HJ Chan, Angela SL Pang, Yvonne LE Ang, Joan RE Choo, Joline SJ Lim, Jun Liang Teh, Aung Lwin, Yuen Soon, Asim Shabbir, Wei Peng Yong, and Jimmy BY So

Institution: National University Health System

### Background

Adding ip paclitaxel (IP-PTX) to paclitaxel/5-fluoropyrimidine has shown promising results in GCPM but has not been studied with platinum/fluoropyrimidine. Our aim was to evaluate IP-PTX with capecitabine/OXA (XELOX) in GCPM.

### Methods

A total of 44 patients with GCPM received IP PTX, oral capecitabine, and intravenous OXA in 21- day cycles. Patients underwent conversion surgery if they had good response to chemotherapy, conversion to negative cytology and no peritoneal disease during surgery. The primary endpoint was overall survival and secondary endpoints were progression-free survival and safety. Outcomes from the trial were compared against 39 GCPM patients receiving systemic chemotherapy (SC) comprising platinum/fluoropyrimidine.

### Results

The median OS for the IP and SC groups was 14.6 and 10.6 months (HR 0.44; 95% CI, 0.26-0.74; P=0.002). The median PFS for the IP and SC group was 9.5 and 4.4 months respectively (HR 0.39; 95% CI, 0.25-0.66; P<0.001). Patients in the SC group were younger (IP vs. SC, 61 vs. 56 years, p=0.021) and had better performance status (ECOG 0, IP vs. SC, 47.7% vs. 76.9%, p=0.007) compared to the IP cohort. In IP group, conversion surgery was performed in 36.1% (13/36) of patients, with a median OS of 24.2 (95% CI 13.1 – 35.3) months, and 1-year OS of 84.6%.

### Conclusion

IP PTX+XELOX is a promising treatment option for GCPM patients. In patients with good response, conversion surgery was feasible with favorable outcomes.

**Abstract GC-04**

## ROLE OF ADDITIONAL STAINING IN THE ASSESSMENT OF THE PERITONEAL REGRESSION GRADING SCORE (PRGS) IN PERITONEAL METASTASIS OF GASTRIC ORIGIN

Authors: Wiebke Solass (1,2), Giorgi Nadiradze (2,3), Marc A Reymond (2,3), and Hans Bösmüller (1,2)

Institution: 1 Institute of Pathology and Neuropathology, and 2 National Center for Pleura and Peritoneum, University of Tuebingen, Germany 3 Department of General and Transplant Surgery, University Hospital Tuebingen, Eberhard-Karls-University Tuebingen

### Background

The PRGS is a four-tied histological regression grading score for determining the response of to chemotherapy. Peritoneal biopsies in every abdominal quadrant are recommended. Therapy response is defined as a decreasing or stable mean PRGS between two therapy cycles. The added value of PAS and Ber-EP4 staining over HE staining for diagnosing PRGS1 (absence of vital tumor cells) is unclear.

### Methods

Retrospective study of peritoneal samples from patients with PM of gastric origin under PIPAC therapy at the University Hospital in Tuebingen between June 2016 and December 2018. Formalin fixed paraffin embedded (FFPE) peritoneal biopsies were scored in three sequential steps). 1) PRGS was set on H&E stain. 2) All biopsies categorized as PRGS 1 or indefinite were reevaluated with PAS stain. 3) All remaining biopsies categorized as PRGS 1 or indefinite were stained with Ber- EP4. This sequential process resulted in the final scoring/assessment of PRGS.

### Results

A total of 339 biopsies obtained during 76 laparoscopies in 33 patients with PM of gastric cancer were analyzed. Biopsies classified as PRGS 1 (no residual tumor, n=95) or indefinite (n=50) were stained with PAS. Tumor cells were detected in 28/145 biopsies (19%). The remaining 117 biopsies were immunostained with Ber-EP4. Tumor cells were detected in 22 biopsies (19%). In total, additional staining allowed detection of residual tumor cells in 50/3 – truncated at 1,500 characters

**Abstract GC-05**

## SIMULTANEOUS INTRAPERITONEAL CISPLATIN CHEMOTHERAPY MAY BE CONSIDERED AS A TREATMENT FOR FAR ADVANCED GASTRIC CANCER WHO UNDERGONE SURGERY

Authors: Eunju Lee, M.D.1, So Hyun Kang, M.D.1, Sangjun Lee, M.D.1, Yongjoon Won, M.D.1, Young Suk Park, M.D.1,2, Sang-Hoon Ahn, M.D.1,2, Yun-Suhk Suh, M.D., Ph.D.1,2, and Hyung-Ho Kim,M.D., Ph.D.1,2

Institution: 1 Department of Surgery, Seoul National University Bundang Hospital, Seongnam, Korea,

2 Department of Surgery, Seoul National University College of Medicine, Seoul, Korea

### Background

Peritoneal seeding is the most common type of recurrence and cause of death for gastric cancer (GC). Nevertheless, there is no established consensus for advanced gastric cancer (AGC) with peritoneal seeding. This study aimed to analyze the effect of ip cisplatin chemotherapy on the survival rate and oncological results of AGC patients who have undergone surgery.

### Methods

Patients who underwent laparoscopic surgery including radical section, palliative surgery and ip chemotherapy for primary gastric adenocarcinoma in our institution were retrospectively reviewed. In ip chemotherapy group, 100mg of cisplatin was administered intraperitoneally. Primary endpoint was survival rate and incidence of early postoperative complication.

### Results

From January 2014 to December 2018, a total of 1373 patients underwent laparoscopic surgery, of which 50 were treated with IP cisplatin chemotherapy. After PSM, each group comprised of 32 patients. There was no significant difference in the early postoperative complications between the two groups (conventional group 12.3%, ip chemotherapy group 10%, p=0.786). There was no statistical difference in overall survival between two groups (p=0.75). In subgroup analysis, median survival was 15.7±12.8 months in the conventional group and 18.6±12.2 months in the ip chemotherapy group in stage IV GC.

### Conclusion

This study showed that simultaneous ip cisplatin chemotherapy seemed to be considered treatment option for AGC.

**Abstract GC-06**

## A CASE REPORT OF EARLY RESPONSE IN A PATIENT UNDERGOING PRESSURIZED INTRAPERITONEAL AEROSOL

**CHEMOTHERAPY (PIPAC) WITH OXALIPLATIN IN COMBINATION WITH IV NIVOLUMAB**

Authors: Hon Lyn TAN, Guowei KIM, Raghav SUNDAR, Cheng Ean CHEE, Asim SHABBIR, Boon Cher GOH, Lingzhi WANG, Jeffrey HY LUM, Jimmy BY SO, and Wei Peng YONG

Institution: National University Health System, Singapore

### Background

PIPAC is a laparoscopic ip chemotherapy delivery technique aiming to improve drug distribution and tissue penetration to treat peritoneal metastases. Intravenous (IV) nivolumab is approved for treatment of progressive gastric cancer after conventional chemotherapy. PIPAC in combination with nivolumab may improve immune activation and response to immune checkpoint inhibition for patients with peritoneal disease.

### Methods

Since June 2020, we have recruited 6 patients with PM in our Phase I study of PIPAC OXA in combination with IV nivolumab (PIANO trial). This trial involves 6- weekly PIPAC OXA and 2-weekly nivolumab. We report a case which has shown early on to have good response to the study treatment.

### Results

The patient is a 68-year-old female diagnosed 35 months ago with poorly differentiated signet ring cell gastric cancer. She underwent R0 D2 radical gastrectomy and was found on table to have peritoneal metastases comprising two deposits. Biomarker analyses showed the tumor to be HER2 negative, p-MMR, MSS, TMB 0, with a CPS score of 0. She received several lines of systemic therapy (TS-1, XELOX, ramucirumab/paclitaxel, and PRL3zumab) prior to joining the PIANO trial. After 1 cycle of PIPAC OXA and 4 cycles of nivolumab, her peritoneal cancer index (PCI) had decreased from 31 to 12. Ascites volume had decreased from 1100mLs (atypical cells on cytology)- TRUNCATED AT 1500 characters

**Abstract GC-07**

## CONVERSION SURGERY IN PATIENTS WITH LIMITED PERITONEAL METASTASIS FROM GASTRIC CANCER TREATED WITH SYSTEMIC CHEMOTHERAPY AND PIPAC: WESTERN CENTER EXPERIENCE

Authors: F. Casella, G. Brancato, M. Bencivenga, C. Ridolfi, C. Puccio, M. Cavallo, and G. de Manzoni

Institution: General and Upper GI Surgery, University of Verona, Italy

### Background

In this study we want to evaluate the role of PIPAC in association to conversion Surgery for patients with limited PM from GCPM.

### Methods

A retrospective analysis of a prospectively collected PIPAC database at upper GI surgery of Verona University was queried for cases with limited PM from GC (PCI ≤ 6) who received systemic chemotherapy plus PIPAC before radical surgery. Surgical and oncological short-term outcomes are reported.

### Results

From December 2019 to May 2021, we recorded 5 cases of conversion surgery for gastric cancer after systemic chemo plus PIPAC. Systemic chemo was FOLFOX in 3 cases and FLOT in 2, while the number of PIPAC procedures was 2 in 2 patients and 1 in the remaining cases. All patients underwent total gastrectomy with D2 lymphadenectomy and HIPEC was added in 3 cases. R0 was achieved in 4 patients; the remaining one had R1 resection. A complete pathological response was obtained in 1 case. No major complications (CD ≥3b) occurred nor after radical surgery or after every PIPAC. The median OS was 16 months. Of note, at 18 months, the patient with complete response is still disease free.

### Conclusion

Available data are not consistent enough to demonstrate the effectiveness of PIPAC in “neoadjuvant” treatment of limited PM from GC. However, our results suggest possible benefit.

**Abstract GC-08**

## BIDIRECTIONAL APPROACH WITH PIPAC AND SYSTEMIC CHEMOTHERAPY FOR PATIENTS WITH SYNCHRONOUS GASTRIC CANCER PERITONEAL METASTASES (GCPM) NOT ELIGIBLE FOR RADICAL SURGERY

Authors: F. Casella, G. Brancato, M. Bencivenga, C. Ridolfi, C. Puccio, M. Cavallo, and G. de Manzoni

Institution: General and Upper GI Surgery, University of Verona

### Background

The aim of this study is to evaluate the efficacy of PIPAC in combination with systemic chemotherapy as bidirectional approach for gastric cancer patients with peritoneal metastases.

### Methods

A retrospective analysis of a prospective PIPAC database was queried for patients with gastric cancer peritoneal metastasis not eligible for surgery who underwent PIPAC with cisplatin and DXR and systemic therapy between February 2019 and May 2021. Surgical and oncological short-term outcomes are reported. The PRGS was used to assess the response.

### Results

A total of 28 PIPAC in 21 patients were performed during systemic chemotherapy. A total of 14 patients underwent 1 PIPAC, 6 patients 2 PIPAC, and one patient 3 PIPAC. Median PCI was 27 (13-39 range). A total of 12 patients received FOLFOX scheme, 6 patients FLOT, 2 patients TCF, and one patient XELOX; 5 patients received also a second line treatment with ramucirumab–paclitaxel before first PIPAC. The overall morbidity of PIPAC was 11% with major complications (CD ≥3a) in one procedure (3%). All the patients completed the planned chemotherapy schedule. PRGS 1 was found in 2 patients, PRGS 2 in 4 patients, PRGS 3 in one patient. The median OS was 14.5 months.

### Conclusion

In our experience the bidirectional therapy with systemic chemotherapy and PIPAC could be considered a safe and effective therapeutical strategy in treatment of unresectable gastric cancer patients with synchronous peritoneal metastases.

**Abstract GC-09**

## PERITONEAL REGRESSION GRADING SCORE (PRGS) IN BIOPSIES FROM CLIPS MARKED PERITONEAL METASTASES COMPARED TO BIOPSIES FROM THE ELEMENT WITH VISUALLY MOST MALIGNANT FEATURES DURING REPEATED PRESSURIZED INTRAPERITONEAL AEROSOL CHEMOTHERAPY (PIPAC)

Authors: Fallah M, Ainsworth AP, Detlefsen S, Fristrup CW, Mortensen MB, Pfeiffer P, and Graversen M

Institution: Odense PIPAC Center, Odense University Hospital, J.B. Winsloews Vej 4, 5000 Odense,Denmark

### Background

PRGS is used for histological response evaluation in PM treated with PIPAC. PRGS is validated, but there is no consensus regarding biopsy strategy. We compared the PRGS in biopsies from clips marked PM to biopsies from PM with visually most malignant features (worst PM).

### Methods

Prospective descriptive study on colorectal PM had OXA based PIPAC, PM from other primaries had PIPAC with cisplatin and DXR. Quadrant biopsies were marked with metal clips. An external surgical oncologist defined the worst PM at the second PIPAC. Worst PM and clips marked PM were biopsied. Biopsies were evaluated by one blinded pathologist.

### Results

Thirty patients were included from March 2020 to May 2021. PM origin: stomach (12), colorectal (8), pancreas (5), and others (5). Median age 64 years (47–79). Patients were pretreated with systemic chemotherapy, 18 patients had bidirectional treatment. Mean PRGS based on clips marked PM at PIPAC 1 and PIPAC 2 was 2.8 (SD 1.0) and 2.1 (SD 0.9). Max PRGS based on clips marked PM at PIPAC 1 and PIPAC 2 was 3.3 (SD 1.2) and 2.6 (SD 1.2). Mean PRGS from the worst PM (PRGS worst) at PIPAC 2 was 2.4 (SD 1.3). There was agreement between PRGS max and PRGS worst in 19 patients. PRGS max was higher in eight patients and lower in three patients compared to PRGS worst (p<0.05).

### Conclusion

Biopsies from clips marked PM did not overestimate treatment response compared to biopsies from PM with visually worst malignant feat – TRUNCATED AT 1,500 CHARACTERS

**Abstract GC-10**

## CYTOREDUCTIVE GASTRECTOMY WITH SIMULTANEOUS PRESSURIZED INTRAPERITONEAL AEROSOLCHEMOTHERAPY (PIPAC) FOR GASTRIC CANCER (GC) WITH LIMITED PERITONEAL METASTASIS (PM). PRELIMINARY RESULTS FROM PHASE-2 STUDY

Authors: Vladimir Khomyakov, Andrey Ryabov, Anna Utkina, Dmitry Sobolev, Sergey Aksenov, Anna Chayka, Alexander Kostrygin, Larisa Bolotina, Olga Kuznetsova, and Andrey Kaprin.

Institution: P.A. Hertsen Moscow Research Oncological Institute – branch of the National Medical

Research Center of Radiology, Moscow, Russia

### Background

Patients with GC and PM have poor prognosis. PIPAC is a new method of ip chemotherapy which is conventionally used as a palliative treatment beyond any SCR. In 2019 we launched the first phase-2 study to assess safety and efficacy of D2-gastrectomy with simultaneous PIPAC in patients with GC and PM.

### Methods

We collected 9 consecutive patients with primary GC and PM who underwent gastrectomy combined with simultaneous PIPAC as conversion surgery after successful systemic chemotherapy ± PIPAC. There were 5 men and 4 women; median age was 56 (from 41 to 72). PCI on operation was 0 in 3 cases, 1-3 – in 6. All patients underwent cytoreductive gastrectomy, combined with simultaneous PIPAC. After finishing open procedure two 10 mm trocars were placed in the right and left flunk and abdomen incision was closured. A 12 mm Hg CO2 pneumoperitoneum was established. The solution of cisplatin 10.5 mg/m2 and DXR 2.1 mg/m2 was aerosolized to the abdomen cavity. The exposure was 30 minutes.

### Results

There were no postoperative deaths and no major complications. We did not detect any negative impact of PIPAC on postoperative recovery after surgery. 2 out of 9 patients died due to progression 9 and 16 months after the start of the treatment. 7 Patients are alive (max – 28 months).

### Conclusion

Cytoreductive gastrectomy with simultaneous PIPAC is safe and feasible alternative to CRS+HIPEC, but further studies are needed to confirm its efficacy.

## Abstract GC-11

**NEOADJUVANT PIPAC IN COMBINATION WITH STANDARD SYSTEMIC CHEMOTHERAPY FOLLOWED BY CONVERSION SURGERY FOR GASTRIC CANCER (GC) WITH LIMITED PERITONEAL METASTASIS (PM)**

Authors: Vladimir Khomyakov, Andrey Ryabov, Anna Utkina, Dmitry Sobolev, Sergey Aksenov, Anna Chayka, Alexander Kostrygin, Larisa Bolotina, Olga Kuznetsova, and Andrey Kaprin.

Institution: P.A. Hertsen Moscow Research Oncological Institute – branch of the National Medical Research Center of Radiology, Moscow, Russia

### Background

Patients with GC and PM have poor prognosis. Complete cytoreduction combined with systemic ± ip chemotherapy is the only curative treatment in selected cases. Neoadjuvant bidirectional chemotherapy is a novel approach for treatment of GC with limited PM.

### Methods

We collected 16 consecutive patients with primary GC and PM who underwent gastrectomy as conversion surgery after successful downstaging as result of upfront systemic chemotherapy and at least one PIPAC session. Selection criteria include resectable primary and peritoneal lesions, limited PM (PCI≤ 6 or Cyt+), no progression or downstaging after neoadjuvant treatment. There were 9 men and 7 women. Median age was 54 y.o (range 35–72). PCI on operation was 0 in 8 cases, 1-3 – in 6, >3 – in 2. All patients underwent cytoreductive gastrectomy, combined with simultaneous HIPEC (n=6) or PIPAC (n=6) in 12 cases.

### Results

There were no postoperative deaths and no major complications. Complete cytoreduction (R0/CC-0) was achieved in 14 cases. A total of 7 out of 16 patients died due to progression. Median survival was 22 months, 2-year survival – 46%.

### Conclusion

Neoadjuvant PIPAC in combination with standard systemic chemotherapy followed by conversion surgery for GC with limited PM provides better response rate, has no impact on postoperative morbidity and demonstrate promising results in selected cases.

## Abstract GC-12

**SAFETY AND EFFICACY OF INTRAPERITONEAL PACLITAXEL PLUS SYSTEMIC FOLFOX FOR GASTRIC CANCER WITH PERITONEAL METASTASIS**

Authors: So Hyun Kang, Sa-Hong Min, Jin Won Kim, Eunju Lee, Sangjun Lee, Hyeon Jeong Oh, Young Suk Park, Yoon Jin Lee, Ji Won Kim, Sang-Hoon Ahn, Yun-Suhk Suh, Hye Seung Lee, and Hyung-Ho Kim

Institution: Seoul National University, Bundang Hospital, Seoul, Korea

Purpose:

This study is a dose-escalation study of ip paclitaxel combined with intravenous fluorouracil, leucovorin, and OXA (mFOLFOX6) to determine the recommended dose in gastric cancer patients.

### Methods

Patients with potentially resectable gastric adenocarcinoma having peritoneal metastasis were enrolled. PCI score was evaluated, and ip chemoport and intravenous chemoport insertion was done. ip paclitaxel was given with an initial dose of 40mg/m2. Target dose was 100mg/m2. Intravenous mFOLFOX6 was administered on the same day at the standard dose. Dose limiting toxicity (DLT) was defined as leukopenia ≥ grade 4, thrombocytopenia ≥ grade 3, febrile neutropenia ≥ grade 3, and other nonhematologic toxicity ≥ grade 3.

### Results

Fifteen patients were enrolled, and two patients were dropped due to consent withdrawal. There was no DLT at 40 and 60mg/m2 doses. Two patients had grade 3 febrile neutropenia at dose

80mg/m2, and thus the final recommended dose was 60mg/m2. Other patients underwent ip paclitaxel and mFOLFOX6 without serious adverse events. Among 5 patients who had second-look diagnostic laparoscopy, 4 patients had a decrease in PCI score. Cytology was converted to negative in 4 out of 5 patients (80.0%). Three patients received total gastrectomy after an average of 8.3 cycles.

### Conclusion

The biweekly regimen of ip paclitaxel and mFOLFOX6 is safe and the recommended dose for a phase II trial is 60mg/m2.

## Abstract GC-13

**A DEDICATED CLINICAL PATHWAY FOR EARLY SIMULTANEOUS CARE TO FORESEE AND OPTIMIZE THE GENERAL CONDITIONS OF PATIENTS WITH UNRESECTABLE GASTRIC PERITONEAL METASTASES TREATED WITH PIPAC**

Authors: G. Brancato, F. Casella, C. Ridolfi, M. Bencivenga, S. Giacopuzzi, F. Blasa, G. Stefani, C. Puccio, M. Cavallo, and G. de Manzoni

Institution: General and Upper GI Surgery, University of Verona, Italy

### Background

GCPM is one of the most common patterns of metastases with a very poor prognosis and scarce quality of life (QoL) in terms of malnutrition, lower control of the pain, malignant ascites, and intestinal occlusion which lead to frequently hospitalization.

### Methods

At Verona University Hospital a dedicated team for the global care of patients with GCPM was recently constituted, including surgical, medical and radiation oncologists, anesthesiologists specialized on pain control as well as psychologist, nutritionists, dedicated nurses and case managers. The main aim of such team is to optimize the general conditions of patients in order to increase the feasibility and tolerability of etiological therapies and improve global QoL in early simultaneous care.

### Results

As soon as a patient is diagnosed with GCPM the early palliative care team is activated. The first step is the simultaneous evaluation of the patient’s care needs: a dedicated score that takes into account the stage of GCPM, the patient’s comorbidities and functional scales and specific symptoms is adopted. The surgeon plans PIPAC in case of symptomatic malignant ascites, while the oncologist evaluates the chance of administering chemotherapy.

### Conclusion

PIPAC is not only a useful procedure to improve the control of ascites but it is essential in the management of early simultaneous care in patients with GCPM.

## Abstract GC-14

**INITIAL EXPERIENCE WITH THE USE OF THE PRESSURIZED INTRAPERITONEAL AEROSOL CHEMOTHERAPY (PIPAC) FOR THE MANAGEMENT OF UNRESECTABLE PERITONEAL METASTASIS IN A NEWLY ESTABLISHED CENTER**

Authors: Mahdi Yahya Alzamanan, Saad Mohammed Almowallad, Taher Abdullah Mahnashi, Abeer Hameed Aljahdali, Abdulbari Mohammed Alawadhi, Samer Ali AlAmmari, Abdullah AlQattan, Mashhour Hussein AlQannas, Mohammed Saleh Alyami, and Delia Cortes Guiral.

Institution: King Khalid Hospital, Najran, Saudi Arabia.

### Background

PIPAC is a recent approach with promising results for patients with PM. We aimed to evaluate survival and postoperative outcome of patients with unresectable PM from different origins treated with chemotherapy and PIPAC in newly established center in the Middle East.

### Methods

A retrospective analysis of a prospective database was queried for all patients diagnosed with unresectable PM who underwent PIPAC in King Khalid hospital, Najran, Saudi Arabia. PIPAC with Cisplatin 10.5 mg/m2 and DXR 2.1 mg/m2 was used for gastric and ovarian PM and oxaliplatin 92mg/m2 was given for colorectal PM, administrated over 30 min at 6-week intervals. Outcome criteria were overall survival and adverse events according to (CTCAE) version4.0.

### Results

Forty-six PIPAC were done for 16 patients. PM was from colorectal, gastric, and ovarian, in 5, 9, and 2 patients, respectively. Thirteen (81.3%), 10 (62.5%), and 4 (25%) patients underwent a second, third, and fourth, PIPAC, respectively. Median PCI was 24.5 (10-39). All patients had concomitant systemic chemotherapy. Median consecutive PIPAC procedures were 2 (1-4). Overall and major complications (CTCAE - III, IV) occurred in 3 (6.5%) and 1 procedures (2.1%) and no mortality. Median overall survival is 9 (2-30) months and 9 (56.2%) of patient still in life on active treatment.

### Conclusion

PIPAC is safe and feasible in association with systemic chemotherapy for unresectable PM in newly established center.

## PHARMACOLOGY, TECHNOLOGY FOR LOCOREGIONAL CHEMOTHERAPY

**Abstract PT-01**

**INTRAVESICULAR TEMPERATURE CHANGE DURING CRS+HIPEC AS PROGNOSTIC FACTOR FOR DFS AND EARLY RECURRENCE**

Authors: Dan Assaf, Amina Issa, Mohammad Adileh, Eyal Mor, Michael Goldenshluger, Shachar Laks, Haggai Benvenisti, Aviram Nissan, and Almog Ben-Yaacov

Institution: Chaim Sheba Medical Center, Ramat-Gan, Israel

### Background

HIPEC has become the most popular method of ip delivery system, with enhanced cytotoxic effect mediated by hyperthermia. However, exaggerated core body temperature (CBT) may increase postoperative complications. The aim of the present study is to examine the influence of body temperature incline on disease free survival and complication, specifically the intravesical temperature (IVT).

### Methods

A retrospective analysis of a prospectively maintained peritoneal surface malignancy database (n=182) was carried out between April 2013 to September 2018. Continuous measurement of IVT was performed, yielding an approximation of linear slope for IVT and dispersion. We compared patients with early recurrence (n=60) and one-year disease free survivor (n=98) for demographics, perioperative, and temperature.

### Results

A total of 158 patients were included in the study, no difference in demographic was noted between the group; however the early recurrence group had more severe postoperative complications. Median follow up was 28.1 months with median DFS of 19.2 months. IVT slope was found to be independently significantly associated with early recurrence (OR=135.5, p=0.008) and worse DFS (HR=8.6, p=0.021).

With a median DFS of 18.4 months, forty-eight of them had an early first year recurrence (38.1%). The same association was noted after stratification for colorectal cancer (CRC) origin (HR=11.3, p=0.016), however there was not significant association with OS.

### Conclusion

TRUNCATED AT 1500 characters

## Abstract PT-02

**EVALUATION OF THE ENVIRONMENTAL CONTAMINATION AND EXPOSURE RISK IN MEDICAL/NON-MEDICAL STAFF AFTER OXALIPLATIN-BASED PRESSURIZED INTRAPERITONEAL AEROSOL CHEMOTHERAPY (PIPAC SECURE)**

Authors: Marion Larroque1, a, b, c, Carine Arnaudguilhem1,c, Brice Bouyssierec, François Quenetb,f, Nabila Bouazzad, Marta Jarliere, Sonia Boulabasd, Sandra Mounicouc, and Olivia Sgarburab,f*

Institution: A Translational Research Unit, Cancer Institute of Montpellier, Montpellier, France bIRCM, Institut de Recherche en Cancérologie de Montpellier, INSERM U1194, Université de Montpellier, Institut régional du Cancer de Montpellier, Montpellier, F-34298, France. c University of Pau and Pays de l’Adour, E2S UPPA, CNRS, Institut des Sciences Analytiques et de Physico-Chimie Pour l’Environnement et les Matériaux (IPREM), UMR5254, Hélioparc, 64053 Pau, France dScientific Direction, ClinicalResearch Center, Cancer Institute of Montpellier, Montpellier, France eBiometrics Unit, Cancer Institute of Montpellier, Montpellier, France fDepartment of Surgical Oncology, Cancer Institute of Montpellier, University of Montpellier, Montpellier, France 1 These authors had equal contributions to the work

### Background

PIPAC is a technique to deliver cytotoxic drugs in the abdomen for the treatment of peritoneal metastases. Pressurization improves the treatment efficacy but increases the risk of exposure for the medical/nonmedical staff.

### Methods

The aim of this study was to evaluate the risk of contamination for the medical/nonmedical staff (nurses, surgeons, anesthesiologists, and cleaning personnel; n=13) during PIPAC with OXA performed according to the French protocol. Blood samples were collected 1 hour before and immediately after PIPAC, and urine samples 1 hour before, and then 3 hours and the morning after PIPAC. In the control, non-exposed group (n=7), only one urine and blood sample were collected. Surface contamination in the operating room was assessed in water and surfanios-impregnated wipe samples. The total elemental platinum in each sample was quantified by inductively coupled plasma mass spectrometry, using a method adapted to quantify trace amounts (ng.L-1) in very low volumes (100μl).

### Results

No surface contamination was detected. Although 25% of urine samples in the exposed group contained platinum, no statistical difference was observed in urine and plasma samples collected before and after PIPAC and with the control group samples.

### Conclusion

These findings suggest that the French PIPAC protocol does not increase the risk of exposure to platinum in all staff categories involved. This protocol could be considered in future occupational policies and consensus statements.

## Abstract PT-03

**INTRAVESICULAR TEMPERATURE CHANGE DURING CRS+HIPEC AS PROGNOSTIC FACTOR FOR DFS AND EARLY RECURRENCE**

Authors: Dan Assaf, Amina Issa, Mohammad Adileh, Eyal Mor, Michael Goldenshluger, Shachar Laks, Haggai Benvenisti, Aviram Nissan, and Almog Ben-Yaacov

Institution: Chaim Sheba Medical Center, Ramat-Gan, Israel

### Background

HIPEC has become the most popular method of ip delivery system, with enhanced cytotoxic effect mediated by hyperthermia. However, exaggerated core body temperature (CBT) may increase postoperative complications. The aim of the present study is to examine the influence of body temperature incline on disease free survival and complication, specifically the IVT.

### Methods

A retrospective analysis of a prospectively maintained peritoneal surface malignancy database (n=182) between April 2013 to September 2018. Continuous measurement of IVT was performed, yielding an approximation of linear slope for IVT and dispersion. We compared patients with early recurrence (n=60) and one-year disease free survivor (n=98) for demographics, perioperative and temperature.

### Results

158 patients were included in the study, no difference in demographic was noted between the group; however the early recurrence group had more severe post-operative complications. Median follow up was 28.1 months with median DFS of 19.2 months. IVT slope was found to be independently significantly associated with early recurrence (OR=135.5, p=0.008) and worse DFS (HR=8.6, p=0.021). With a median DFS of 18.4 months, forty-eight of them had an early first year recurrence (38.1%). The same association was noted after stratification for CRC origin (HR=11.3, p=0.016), however there was not significant association with OS.

### Conclusion

Truncated at 1,500 characters

## Abstract PT-04

**A PHASE 1 DOSE ESCALATING STUDY PIPAC WITH OXALIPLATIN, CISPLATIN AND DOXORUBICIN IN PERITONEAL METASTASIS**

Authors: Manuela Robella, Michele De Simone, Paola Berchialla, Monica Argenziano, Alice Borsano, Shoeb Ansari, Ornella Abollino, Eleonora Ficiarà, Armando Cinquegrana, Roberta Cavalli, and Marco Vaira

Institution: Candiolo Cancer Institute, FPO – IRCCS

### Background

PIPAC is a new laparoscopic ip chemotherapy approach with the advantage of a deeper tissue penetration. Thus far, OXA has been administered at an arbitrary dose of 92 mg/m2, cisplatin (CDDP) at 7.5 mg/m2 and DXR 1.5 mg/m2.

### Methods

This is a phase I 3+3 dose escalation study aimed at identifying the maximum tolerable dose of the three drugs. The starting dose of OXA was 100 mg/m2. CDDP was used with DXR: 15 mg/m2 and 3 mg/m2 were the respective starting doses. Safety was assessed according to CTCAE.

### Results

Thirteen patients were submitted to 1 PIPAC, 7 patients were treated with CDDP and DXR and 6 patients with OXA; no dose limiting toxicities and major side effects were found. Common adverse events included postoperative abdominal pain and nausea. The maximum tolerable dose was not reached. The highest dose cohorts (OXA 135 mg/m2; CDDP 30 mg/m2 and DXR 6 mg/m2) tolerated PIPAC well. A further 4 patients underwent repeated PIPAC as off-label treatment with CDDP 30 mg/m2 + DXR 6 mg/m2: no postoperative morbidity or cumulative toxicities were reported. Serological analyses revealed no trace of DXR in any dose level. Serum levels of cisplatin and OXA reached a peak at 60–120 min after PIPAC and were still measurable in the circulation 24 hours after the procedure.

### Conclusion

CDDP and DXR may be safely used as PIPAC at a dose of 30 mg/m2 and 6 mg/m2, respectively; OXA can be used at 135 mg/m2. The dosages achieved to date are the highest ever used in PIPAC.

## Abstract PT-05

**NEW DEVICE FOR PRESSURIZED INTRAERITONEAL AEROSOL CHEMOTHERAPY (PIPAC) – FIRST CLINICAL EXPERIENCE**

Authors: David Hoskovec1, Radim Skala2, and Zdenek Krska1

Institution: 1 General University Hospital - 1st Department of Surgery, 2 Skala Medica s. r. o., Czech Republic

### Background

PIPAC is a promising technique for ip delivery of chemotherapy. There has been only one device on the medical market, but several companies are working on their own device. We know about the German prototype named Prototype4 and the Korean prototype.

### Methods

Czech company Skala Medica s.r.o. developed original Czech product for PIPAC (and PITAC) miniinvasive surgical atomizer MCR-4 TOPOL®. This device is patented and has EC certification.

### Results

MCR-4 TOPOL® is 216 mm long and the diameter is 8 mm. The diameter of the jet is 0.35 mm and the spray angle is about 80°. The body of the atomizer is securely connected with a high-pressure tube. The working pressure is 100 – 330 psi. MCR-4 TOPOL® was tested in the laboratory for spray pattern, size and speed of drops. Another test was done to check the compatibility of MCR-4 TOPOL® with laparoscopy equipment MCR-4 TOPOL® was first clinically used in General University Hospital in Prague after obtaining the patent and EC certification. The spray pattern is symmetric. The speed of drops of the spray depends on the distance between the end of the device and the boundary of the sprayed space. The same is possible to say about drop size. The second factor is radial distance.

### Conclusion

Clinical application in the first three patients was without any technical or medical complications. MCR-4 TOPOL® is the second device for PIPAC/PITAC on the market.

## Abstract PT-06

**PRESSURIZED INTRAERITONEAL AEROSOL CHEMOTHERAPY (PIPAC) NEBULIZER MANDATORY TESTING: TECHNICAL NOTE & ALGORITHM**

Authors: Delgadillo Xavier^1^ Wüthrich Philippe^2^ and Gaspar Tunde^1^

Institution: ^1^Center Médico Chirurgical Volta, La Chaux de Fonds, Switzerland. ^2^Center Hospitalier Genolier, 1273- Arzier, Switzerland.

### Background

The PIPAC procedure for the treatment of PM is nowadays almost worldwide practiced. Despite the instruction courses implemented by authorities, pitfalls may happen just before or during the procedure.

### Methods

Preventive pitfalls have been analyzed after an initial observational phase of this study, and we had observed that the Capnopen® device’s failure before the aerosolization of chemotherapy drugs planned for the PIPAC can be preventively detected following an exclusion nebulizer’s defect algorithm.

### Results

After an exhaustive PubMed, Google Scholar, and Scopus research, with the key seeking words: “PIPAC failure” and “nebulizer PIPAC miscarrying” we could not find any published report on the international literature concerning the most worldwide nebulizer device used, Capnopen® directly related to the product malfunction, or any patient’s complications due to the device’s impairment. We had directly contacted the manufacturer, to obtain technical and specific details of manipulation and use of the nebulizer and we describe some important rules to prevent any hazardous situation related to a possible device’s internal defect. Finally, we present a flow chart, to apply just before any use of any nebulizer before a PIPAC procedure.

### Conclusion

It is very useful to have a flow chart algorithm to follow a nebulizer’s test just before any PIPAC procedure, preventing any malfunction event.

## Abstract PT-07

**ENHANCED INTRAPERITONEAL DELIVERY OF CHARGED, AEROSOLIZED CURCUMIN NANOPARTICLES BY ELECTROSTATIC PRECIPITATION**

Authors: Arianna Castagna, Alexandra J Zander, Iaroslaw Sautkin, Marc Schneider, Ranjita Shegokar, Alfred Königsrainer, and Marc André Reymond

Institution: Department of General, Visceral & Transplant Surgery, Comprehensive Cancer Center, University of Tübingen, Hoppe-Seyler-Strasse 3, Tübingen 72076, Germany; Department of Pharmacy, Biopharmaceutics & Pharmaceutical Technology, Saarland University, Saarbrücken 66123, Germany; Capnomed GmbH, Albring 81, Zimmern o.R. 78658, Germany.

### Aims

To investigate the potential of curcumin-loaded polylactic-co-glycolic acid nanoparticles (CUR-PLGA-NPs), alone and with electrostatic precipitation, for improving tissue uptake during PIPAC.

### Methods

Positively and negatively charged CUR-PLGA-NPs were delivered as PIPAC into inverted bovine urinary bladders ex vivo. The experiment was repeated with the additional use of electrostatic PIPAC.

### Results

Positively charged CUR-PLGA-NPs increased depth of tissue penetration by 81.5% and tissue concentration by 80%. Electrostatic precipitation further improved the uptake of positively charged CUR-PLGA-NPs by 41.8%.

### Conclusion

The combination of positive charge and electrostatic precipitation has significant potential to improve tissue uptake of nanoparticles during ip chemotherapy.

## Abstract PT-08

**WHAT IS THE MINIMAL APPLICATION TIME FOR ELECTROSTATIC PRESSURIZED INTRAPERITONEAL AEROSOL CHEMOTHERAPY (EPIPAC)?**

Authors: Iaroslav Sautkin, Arianna Castagna, Alfred Königsrainer, Giorgi Nadiradze, and Marc Reymond

Institution: University of Tübingen, University Hospital Tübingen, Department of General, Visceral and Transplantation Surgery, National Center for Pleura and Peritoneum

### Background

Electrostatic precipitation (EP) improves PIPAC’s spatial distribution and pharmacological properties. However, the minimal time needed to reach an optimal tissue drug concentration (TC) remains uncertain.

### Methods

Ex-vivo study in inverted bovine urinary bladders (eIBUB model). Aerosolization of 50ml DXR 2.7mg and 150ml cisplatin (CIS) 13.5mg. Five groups (Gr) were compared: I: EP for 6min, II: EP for 10min, III: EP for 30min, IV: EP for 36min, V: control=PIPAC for 36min. Activation of EP at T0min (defined as the start of aerosolization) in groups I & IV, at T6min in groups II & III. Outcome criteria: a) aerosol tissue uptake, b) DOX TC.

### Results

Aerosol absorption into the peritoneal tissue was superior by 177±51% in Gr I (ePIPAC, 6min vs PIPAC 36min), p>0.05. DXR TC after 6min ePIPAC was comparable with 36min PIPAC, p>0.05, and decreased with EP time (ng/mg: Gr I: 2.0±2.5, II: 1.3±1.1, III: 0.6±0.8, IV: 1.00±1.2, V=PIPAC (control): 2.3±1.7; (V vs II, III, IV p<0.05)). Droplets were observed at the outer eIBUB surface at a longer exposure time.

### Conclusion

Aerosol absorption was superior after ePIPAC vs PIPAC. DOX TC after 6min ePIPAC (starting at T0) approaches PIPAC after 36min. DXR TC decreased over time, suggesting the electrostatic field transporting the drug to the outer eIBUB surface after initial tissue uptake. These results should be confirmed in a large animal model before ePIPAC time can be safely decreased to 6 min in human patients.

## Abstract PT-09

**IN VITRO STUDY OF AEROSOL DROPLET IMPACT ON THE PERITONEAL SURFACE AFTERNEBULIZATION USING THE CAPNOPEN DEVICE**

Authors: Mohammad Rahimi-Gorji1,2,3, Stéphane Dorbolo4, Charlotte Debbaut2,3, Ghader Ghorbaniasl5, Wouter Willaert1,3, and Wim Ceelen1,3

Institution: 1Laboratory for Experimental Surgery, Department of Human Structure and Repair, Ghent University, Ghent, Belgium; 2IBiTech – Biommeda, Department of Biomedical Engineering, Ghent University, Ghent, Belgium; 3CRIG – Cancer Research Institute Ghent, Belgium; 4FNRS, GRASP lap, CESAM Research Unit, University of Liège, Liège, Belgium; 5Department of Mechanical Engineering, Vrije Universiteit Brussel (VUB), Brussels, Belgium

### Background

PIPAC is a drug delivery strategy to treat peritoneal cancer. The efficacy of PIPAC ultimately depends on the interaction between the aerosol droplets and the cancerous peritoneal surface. Here, we investigated this interaction using high speed imaging in an experimental setup.

### Methods

The CapnoPen nebulizer was positioned 30 cm above a 50 cm^2^ fragment of porcine abdominal wall with the mesothelial surface facing the nebulizer. Saline solution was nebulized at flow rates of 0.5, 0.8, and 1 mL/s. The impaction of droplets on the surface was imaged in one plane using a highspeed camera (10.000 fps, Phantom MIRO M310). Ten droplets were selected randomly to calculate their average velocities.

### Results

Image analysis showed that the large majority of droplets stick to the surface after impaction. A liquid film was rapidly formed on the tissue surface, which affected the impaction pattern. Some large droplets fragmented after impaction, and were rarely bounced back. Higher flow rates caused more turbulence. The average velocity just before impaction was calculated 2.29, 2.71, and 3.43 m/s for flow rates of 0.5, 0.8, and 1 mL/s, respectively.

### Conclusion

Most droplets stick to the surface after impaction. The average velocity of droplets before impaction increases with flow rate but is much lower compared to the initial velocity. These results may allow improving droplet behavior and tissue interaction during PIPAC.

## Abstract PT-10

**STATISTICAL COMPARISON BETWEEN THE PHYSICAL PROPERTIES OF THE PARTICLES ON STANDARD PIPAC AND ULTRASONIC PIPAC**

Authors: Rafael Seitenfus, Eduardo Dipp de Barros, Ana Carolina Bathelt Fleig, Günther Ayala Pereira, Cassio Bona Alves, Gustavo Andreazza Laporte, Carlos Humberto Cereser junior, Giovanni Luigi, and Carla Joana Hugueney Franco Lobo

Institution: Hospital Santa Casa de Misericordia de Porto Alegre, Oncology Surgery, Porto Alegre, Brazil

### Background

The modality of peritoneal chemotherapy seeks to explore pharmacokinetic properties not possible with the use of systemic chemotherapy. This poster intent is to describe the physical properties of a new modality of aerosolization using ultrasonic energy mechanim 2.

### Methods

The measurement of the size of the aerosol droplets formed was carried out in 7 aerosolizations with H_2_O. The 6 measurements of aerosolization by ultrasound were performed using a patent-pending ultrasonic aerosolization generator. The aerosol rod varied only in the design of the tip or the material the rod was machined from. The two designs of probe tips evaluated were defined as a multidirectional tip and a hat tip.

### Results

The temperature in the 30 minutes of aerosolization ranged from 3 to 5 degrees Celsius (°C) reaching the level maximum of 39°C. The total number of aerosolized droplets measured was 56,649 and average of 9441 aerosolized droplets per measurement. The mean DV 50 was 39.17 μm for all ultrasonic aerosolizations. The comparative analysis was evaluated in 3 different particles size ranges described as shown in Table 1. Ultrasonic aerosolization by hat rod, aluminum multidirectional or titanium multidirectional showed a significant difference when compared to standard PIPAC.

### Conclusion

The ultrasonic aerosolization of therapeutic agents in the peritoneal space is feasible, presents the adequate size of particles, warms the therapeutic agent, and amplifies the time of application. – TRUNCATEDD AT 1500 CHARACTERS

## Abstract PT-11

**EVALUATION OF THE FEASIBILITY OF ULTRASONIC AEROSOLIZATION OF THERAPEUTIC SUBSTANCE IN ANIMAL MODEL**

Authors: Rafael Seitenfus, Eduardo Dipp de Barros, Carlos Cereser Junior, Carla Joana Hugueney Franco Lobo, Giovanni Luigi, Ana Carolina Fleig, and Cassio Bona Alves

Institution: Santa Casa de Misericórdia de Porto Alegre, Porto Alegre, Brazil

### Background

The use of chemotherapy in the peritoneum seeks to explore pharmacokinetic properties not possible with the use of systemic chemotherapy. PIPAC aims to optimize the distribution and depth of penetration of therapeutic agents into the peritoneal space. This form of application uses devices that aerosolize therapeutic substances using high injection pressure atomization. This poster seeks to describe the feasibility in an animal model of a new modality of aerosolization of a therapeutic substance in the peritoneum using ultrasonic energy.

### Methods

Five procedures were performed in porcine models in the premises of the Hospital Israelita Albert Einstein in Sao Paulo, Brazil. All animals underwent videolaparoscopy with 8 mmHg pneumoperitoneum. The ultrasonic device was attached to a trocar and fixed in a mechanical arm and submitted to an ultrasonic aerosolization of 200ml of 2% silver nitrate. Esophageal thermometers were positioned inside the peritoneal cavity.

### Results

Table

### Conclusion

The observed ultrasonic aerosolization time was the most distinctive feature of the traditional aerosolization process. Here, our models used an injection flow of 200 to 400 ml/h and considering the need to change syringes, the time measured ranged from 30 to 48 minutes to aerosolize 200 ml of solution. The temperature in the procedures ranged from 3 to 5ºC, not reaching levels higher than 39°C. These first observations identified distinct characteristics from the tradition.

## Abstract PT-12

**INTRAPERITONEAL NAB-PACLITAXEL: RESULTS FROM THE PHASE I PIPAC STUDY**

Authors: Wim Ceelen, Louis Sandra, Leen Van de Sande, Martin Graversen, An Vermeulen, Dries Reynders, Sarah Cosyns, Anne Hoorens, and Wouter Willaert

Institution: Ghent University, Belgium

### Background

We performed a phase I first-in-human trial of PIPAC using nanoparticle albumin bound paclitaxel (Nab-PTX, Abraxane) in patients with upper gastrointestinal, breast, or ovarian cancer.

### Methods

Eligible patients underwent three PIPAC treatments using Nab-PTX with a four-week interval. The dose of Nab-PTX was escalated from 35 to 140 mg/m2 using a Bayesian approach until the maximally tolerated dose (MTD) was reached. Secondary endpoints included surgical morbidity, pharmacokinetics (PK), histological treatment response, and OS.

### Results

Twenty-three patients were included; thirteen (65%) patients combined PIPAC therapy with systemic chemotherapy. The primary tumor was gastric (55%), ovarian (20%), hepatobiliary or pancreatic (15%), breast (5%), and miscellaneous (5%). No dose limiting toxicity was observed. Eight patients showed surgical site complications including infection and dehiscence (grade 1 to 3). Treatment was associated with histological response in 35% of patients, while stable disease and progressive disease were found in 35% and 30%, respectively. The absorption of PTX continued long after the end of the procedure (30 min), whith the Tmax reached between 2 and 6 h after initiation of the procedure. Tumor PTX concentrations suggested accumulation. European Organization for Research and Treatment of Cancer (EORTC) and Visual Analog Score (VAS) scores remained stable.

### Conclusion

PIPAC with Nab-PTX may be applied safely up to a dose of 140 mg/m2 and results in a favorable PK profile and promising anticancer activity.

## Abstract PT-13

**ENPERITO: EFFECTIVE POLY(ALKYL CYANOACRYLATE) NANOPARTICLES FOR TREATMENT OF PERITONEAL METASTASES**

Authors: Karianne Giller Fleten, Astrid Hyldbakk, Sofie Snipstad, Andreas Åslund, Evelina Folkesson, Yrr Mørch, and Kjersti Flatmark

Institution: Oslo University Hospital, Norway

### Background

PM are associated with poor prognosis, and novel treatment strategies are needed. Drugs administrated ip are rapidly absorbed, limiting the efficacy locally in the peritoneal cavity. We have developed a novel nanoparticle-based drug delivery system (ENPERITO) where drugs are encapsulated in poly(alkyl cyanoacrylate) nanoparticles. The aim is to increase the local drug concentration and prolong the drug residence time in the peritoneal cavity, and to decrease systemic toxicity.

### Methods

Cabazitaxel (CBZ) was administered alone or encapsulated (PACA-CBZ). Biodistribution and uptake of CBZ were investigated after ip and intravenous injection in mice with and without tumors. In addition, mice with peritoneal tumors from ovarian and colorectal cancer were treated with free CBZ or PACA-CBZ.

### Results

Higher concentrations of CBZ were detected in organs and tumors after injection of PACA-CBZ compared to free CBZ, showing that nanoparticle encapsulation resulted in increased drug retention. Treatment experiments in mouse models established from ovarian and colorectal cancer showed increased survival and reduced tumor growth in mice treated with PACA-CBZ compared to mice treated with free CBZ.

### Conclusion

The ENPERITO drug delivery system shows great promise as a novel treatment strategy for peritoneal metastases, where nanoparticle encapsulation resulted in increased drug retention and efficacy.

## BASIC SCIENCE OF PLEURAL AND PERITONEAL DISEASE

**Invited lecture**

**PERITONEAL METASTASIS ASSOCIATED TUMOR MICROENVIRONMENT**

Authors: Wim Ceelen

Institution: Ghent University, Belgium

The pathophysiology of PM remains poorly characterized. Also, the efficacy of current treatment modalities, including surgery and ip delivery of chemotherapy, is limited. Increasingly, therefore, efforts are being developed to unravel the PM cascade and at understanding the PM-associated tumor microenvironment (TME) and peritoneal ecosystem as potential therapeutic targets. During this lecture, I review recent insights in the structure and components of the TME in colorectal PM, and discuss how these may translate into novel therapeutic approaches aimed at reengineering the metastasis-promoting activity of the stroma.

## Abstract BS 01

**INFLUENCE OF INTRAPERITONEAL, EXTRACELLULAR PH ON TUMOR PHENOTYPE**

Authors: QianLu Yang, Frank-Jürgen Weinreich, Giorgi Nadiradze, Rami Archid1, Christoph Trautwein, Stefan Kommoss, Alfred Königsrainer, and Marc A. Reymond.

Institution: National Center for Pleura and Peritoneum, University of Tübingen, Germany

### Background

Cancer cells that shed into the peritoneal cavity need to resist to anoikis and escape from immune elimination. For this purpose, they modify their metabolism to adapt to the hostile environment. There is little data on intracellular pH (pHi), and on the influence on extracellular pH (pHe) on tumor phenotype.

### Methods

Intraoperative measure of ip pHe, electrolytes, lactate, and pO2 and pCO2 in gastric and ovarian cancer vs. control patients. Metabolic assay (MTT) in human gastric (MKN45) and ovarian (OAW42) cell lines with pHe gradient between 6.0 and 7.5. Scratch assay for cell migration (Cell Zen Owl) and apoptosis measured with FACS (FITC) in the same pHe range in both cell lines.

### Results

pHe was significantly higher in cancer patients (7.68 ± 0.33) vs. controls (6.87 ± 0.02), p< 0.001. Metabolic activity *in vitro* increased with pHe in both cell lines (ANOVA, MKN45: p < 0.001, OAW42:

p < 0.001), as did cell migration (OAW42: p < 0.001). An acidic pHe correlated with a higher apoptotic rate.

### Conclusion

Intraperitoneal pHe is more basic in cancer patients vs. controls. *In vitro*, cell survival, migration, and metabolic activity are impaired in the presence of a lower extracellular pHe. Manipulating pHe might be an effective target for the prevention and therapy of peritoneal metastasis.

## Abstract BS-02

**CYTOREDUCTIVE SURGERY AND HIPEC IN AN ENHANCED RECOVERY AFTER SURGERY (ERAS) PROGRAM: A REVIEW OF THE LITERATURE AND A META- ANALYSIS**

Authors: Manuela Robella, Alba Ilari, Paola Berchialla, Veronica Sciannameo, Alice Borsano, Michele De Simone, and Marco Vaira.

Institution: Candiolo Cancer Institute, FPO – IRCCS

Emerging evidence suggests that ERAS program shows a perioperative benefit in patients with peritoneal Metastasis submitted to cytoreductive surgery (CRS) +/- HIPEC. This systematic review and meta-analysis will assess the safety and efficacy of the ERAS program.

A systematic search was done to identify all relevant literature on the ERAS protocol applied in patients submitted to CRS +/- HIPEC published between Jan 1, 2010 and Dec 31, 2020.

A total of 136 articles, reports and clinical studies were identified: 4 studies were selected for CRS and 6 for CRS + HIPEC for a total of 649 and 278 procedures, respectively.

Meta-analysis of the comparative studies showed that the application of ERAS program contributed significantly to shorter hospital stay in patients submitted to CRS (M2D -0.29, 95% CI -0.41; -0.18) and CRS + HIPEC (M2D -0.48, 95% CI -0.77; -0.18). Comparing the two groups in terms of readmission rate, ERAS protocol is associated to a risk ratio of 1.05 in CRS + HIPEC group (95% CI 0.33;3.38) and 0.55 in CRS group (95% CI 0.21;1.41). Pooled analysis of all comparative studies demonstrated lower postoperative morbidity rate in the CRS group: a risk ratio of 0.66 (CI 95% 0.58 – 0.76) has been reported. The analysis wasn’t carried out in HIPEC group due to the lack of data.

ERAS program reduces significantly hospital stay and postoperative morbidity rate in patients submitted to CRS +/-HIPEC for peritoneal metastasis.

## Abstract BS-03

**SYNGENEIC MOUSE MODEL FOR COLORECTAL CANCER PERITONEAL METASTASIS**

Authors: Sam Ernst (1,2,3), Olivier De Wever (2,3), and Wim Ceelen (1,3)

Institution: (1) Laboratory of Experimental Surgery, Department of Human Structure and Repair, (2) Laboratory of Experimental Cancer Research (LECR), Department of Human Structure and Repair, (3) Cancer Research Institute Ghent (CRIG), Ghent University, Ghent, Belgium.

### Background

Because CRC PM is associated with high relapse rates using standard of care treatment, it is crucial to search for a novel treatment strategy. To efficiently study the efficacy or potential of a novel treatment, preclinical models of CRC PM should be used. For potential immunotherapies, a functional immune system is a prerequisite, making syngeneic mouse models a valid option.

### Methods

Two syngeneic mouse models for CRC PM were established by injecting CT26-Luc and MC38-Luc murine colorectal cancer cells into BALB/c mice and C57BL/6 mice, respectively. Two injection Methods were used, namely an ip injection and a subperitoneal (SP) injection with cells dissolved in PBS and Matrigel, respectively.

### Results

For both cell lines, ip injection resulted in multiple m2all tumor nodules found throughout the abdominal cavity. The most common site of tumor formation was the mesentery. In contrary, SP injections resulted in larger tumor nodules at the abdominal wall. ip injections seemed to be more harmful for the mice.

### Conclusion

We established a syngeneic mouse model for CRC PM by injecting murine tumor cells ip or SP. SP injections result in larger, isolated tumor nodules which may be suited to drug penetration studies. ip injections result in a heterogenous PM spread throughout the abdominal cavity and a shorter animal survival.

## Abstract BS-04

**PRGS AND IN-SITU IMMUNOPHENOTYPE AS A COMBINED „TOOL“ FOR MONITORING THE THERAPEUTICAL EFFICIENCY OF PIPAC IN DIFFERENT PERITONEAL CANCER DISEASES: A SINGLE CENTER EXPERIENCE**

Authors: Tarkan Jäger1, Eckhard Klieser^2^, Bettina Neumayer^2^, Ricarda Gruber1, Jan Philipp Ramspott³, Philipp Schredl1, Klaus Emmanuel1, and Daniel Neureiter^2^

Institution: 1 Department of Surgery, Paracelsus Medical University/Salzburger Landeskliniken

(SALK), 5020 Salzburg, Austria 2 Institute of Pathology, Paracelsus Medical University/Salzburger

Landeskliniken (SALK), 5020 Salzburg, Austria 3 Department of Gynecology and Obstetrics, Münster University Hospital, Münster, Germany

### Background

PIPAC is able to induce regression of PM. The PRGS is used for assessment of therapy response. The role of the related immune phenotype is still unknown.

### Methods

The PRGS was judged on four peritoneal biopsies with HE-staining and tumor-entity related immunostainings to quantify infiltrating tumor cells. Additionally, the effect of localized immune response was analyzed by additional immunohistochemical staining for CD3, CD4, CD8, CD25, and TIA, too.

### Results

Overall, peritoneal metastasis of the enrolled 48 patients (female/male: 26/22 with a mean age of 60.3 +/- 11.6 years) derived mainly of gastric cancer, malignant mesothelioma and ovary cancer. Based on a total sum of 134 PIPACs and 532 PRGS the mean [with confidence interval]/median PRGS after the first and after the last PIPAC were 2.6 [2.4-2.7)/3.0 and 2.0 [1.9-2.2//2.0 with the lowest value of 1.1 [0.8-1.4]/1.0 at the fourth PIPAC. Additionally, we were able to link the PRGS and associated fibrosis to a specific regulative immune response.

### Conclusion

We demonstrated that the standardized applied PRGS is adequate to monitor the therapy response and outcome in cases with enhanced PM. The related in-situ immune phenotype supported this notion. In the future, the definitive predictive and prognostic role of the PIPAC induced immune reaction needs to be evaluated.

## Abstract BS-05

**EVALUATION OF DOXORUBICIN-LOADED EXTRACELLULAR VESICLES DELIVERED BY PIPAC IN A MURINE MODEL OF COLONIC PERITONEAL METASTASIS**

Authors: Aurore Protat, Florence Gazeau, Amanda K A Silva, Cherukula Kondarredy, and Marc Pocard.

Institution: Inserm UMR 1275 CAP Paris tech 75010 Paris, CNRS UMR 7057 Laboratoire Matière et Systèmes complexes 75205 Paris Cedex 13. France

### Background

Extracellular vesicles (EVs) are nanocarriers for exogen molecules and have high specificity for cancerous cells. We hypothesis chemotherapy-loaded EV associated to PIPAC delivery could increase the amount of chemotherapy inside nodules. Our objective was to evaluate the feasibility of PIPAC delivering EVs obtained with mesenchymal stem cells, in a murine colonic Metastasis model.

### Methods

DXR-loaded EVs were produced by UMR 7057 laboratory.EV were measured before and after PIPAC. Murine colonic Metastasis was induced by peritoneal injection of CT26 cells in BALB/c mice. EV’s effect on metastasis was compared to free-DXR (ip administration) in forty mice. The primary outcome was the PCI evaluation. Secondary outcomes were percentage of necrosis.

### Results

Numbers of EV were identic before and after PIPAC. The size of EV was 150 nm versus 110 nm before PIPAC procedure. The Doxo concentration is more than 5 lower in EV than in free drug delivery solution. PCI in the control group is 27, different than with free DXR, at 21 (p=0.01) but not different with EV-DXR at 25. 30% of metastasis nodule presented extensive necrosis in control group but 50% with free DXR and 50% with EV.

### Discussion

To our knowledge it is the first description of PIPAC delivering EVs. PIPAC did not alter the EV property as number and size. DXR could be delivered using EVs with PIPAC. Technology to increase DXR concentration in the EV is mandatory to confirm a – truncated at 1,500 characters

## Abstract BS-06

**DEVELOPING A BIOMARKER PANEL TO PREDICT ADJUVANT PLATINUM CHEMOSENSITIVITY IN OVARIAN CANCER**

Authors: Nicholas Brian Shannon, Laura Ling Ying Tan, Qiu Xuan Tan, Joey Wee-Shan Tan, Josephine Hendrikson, Wai Har Ng, Gillian Ng, Ying Liu, Xing-Yi Sarah Ong, Ravichandran Nadarajah, Grace Hwei Ching Tan, Melissa Ching Ching Teo, Khee Chee Soo, Claramae Shulyn Chia, and Chin-Ann Johnny Ong

Institution: National Cancer Center, Singapore

### Background

Platinum resistance in ovarian cancer contributes to tumor recurrence. We aimed to identify a biomarker panel to predict adjuvant platinum sensitivity in ovarian cancer patients.

### Methods

Using the Genomics of Drug Sensitivity in Cancer (GDSC) and The Cancer Genome Atlas (TCGA) databases, we identified 4 biomarkers (CYTH3, GALNT3, S100A14, and ERI1) to predict cisplatin sensitivity. Validation was performed via immunohistochemical (IHC) staining on a tissue microarray (TMA) containing samples from patients treated with surgical resection and adjuvant carboplatin, and assessed for response via the RECIST criteria (n=50). Models were built to predict chemosensitivity and further assessed by their ability to prognosticate patients in datasets from gene expression omnibus (GEO) (n=561 over 3 datasets).

### Results

The area under the ROC curve (AUC) for chemosensitivity prediction in the TMA dataset was 0.7-0.8.

When the model to predict chemosensitivity was applied to the GEO datasets and performance assessed on whether it predicted 2-year overall survival, the AUC for the gene model (AUC 0.59-0.66) was comparable to clinical information alone (tumor grade and stage, AUC 0.58-63).

### Conclusion

Chemosensitivity can be predicted via tumor biology assessment. Moreover, the prediction is clinically significant as it prognosticates overall survival. Clinical information coupled with molecular marker assessment will offer the best chance at optimizing therapeutic decision making.

## Abstract BS-07

**THE CHARM STUDY: A PROSPECTIVE COHORT STUDY HARNESSING A 3-BIOMARKER PANEL FOR MOLECULAR PROGNOSTICATION IN PERITONEAL METASTASIS**

Authors: Chin-Ann Johnny Ong, Ying Liu, Josephine Hendrikson, Gillian Ng, Wen Hui Tu, Xing-Yi Sarah Ong, Joey Wee-Shan Tan, Qiu Xuan Tan, Wai Har Ng, Jolene Si Min Wong, and Claramae Shulyn Chia

Institution: National Cancer Center Singapore

### Background

Intraabdominal fluid contains multiple signalling proteins of biological significance. We conducted a prospective single center study to identify and validate liquid intraabdominal prognostic and therapeutic biomarkers.

### Methods

Using orthogonal proteomics analysis of peritoneal fluid coupled with transcriptomic cellular response in cells exposed to peritoneal fluid and clinical correlation with The Cancer Genome Atlas (TCGA) data, we identified putative paracrine factors that inform biology and clinical outcome of PM patients. Validation was performed via enzyme linked immunosorbent assay (ELISA) and a rapid immunoassay system.

### Results

A total of 112 putative proteins were associated with intra-abdominal fluid from the CTD gene-disease database. Transcriptomic analysis of 2 cell lines treated with intra-abdominal fluid demonstrated upregulated STAT3 signalling. Biological curation of secreted proteins by combining both analyses shortlisted 17 highly prognostic targets when corelated with TCGA data (n=1000+). Finally, we validated a 3-biomarker prognostic panel that outperforms current clinical factors including cytology and PCI (n=149, p=.001). Panel prognostic significance was confirmed via a rapid 3-hour automated immunoassay (n=110).

### Conclusion

We identified and validated a rapid 3-biomarker prognostic immunoassay platform. This allows a rapid molecular stratification strategy to predict response to PIPAC and patient suitability for CRS and HIPEC.

## Abstract BS-08

**PREANALYTICAL SAMPLE PREPARATION FROM OMENTAL MILKY SPOTS FOR MULTICHANNEL FLUORESCENCE ACTIVATED CELL SORTING (FACS)**

Authors: Caroline Graf1, Simone Pöschel2, Frank-Jürgen Weinreich1, A. Königsrainer1,3, Marc A. Reymond1,3, and G. Nadiradze1,3

Institution: 1 National Center for Pleura and Peritoneum, NCT South-West Germany, 2 FACS central facility, Klinkum Berg, 3 Dept. of General and Transplant Surgery, University Hospital, Tübingen, Germany

### Background

Milky spots (MSs) are lymphatic structures occurring with high frequency on the greater omentum. MS are decisive in the preferential implantation of PM. Whereas the MS’ cellular components are known, their phenotypic characterization during the progression of PM remains unclear.

### Methods

We have developed a preanalytical sample preparation technique enabling 12-channel FACSorting of immune cells from the human omentum.

### Results

A Ø 5cm sample of the greater omentum was obtained in the OR, and mechanically mashed through a 70 μM cell strainer. Pellets were resuspended in 10ml PBS/0.5% BSA/2 mM EDTA, layered on 5ml Ficoll, and centrifuged for 30 min at 2000rpm. The interphase mononuclear cells were pipetted off. Cells were resuspended in PBS and centrifuged for 2x10 min (1500rpm and 1300rpm) at RT with a brake. The pellets were stored at -80°C. After thawing, cells were stained with appropriate human antibody panels and costained with the life/dead dye ZombieAqua (BD), washed with PBS/1% BSA, and transferred to FACS tubes. Optimal antibody concentration was determined by titration. Flow cytometry data were acquired using a BD FACSCanto™ II (BD Bioscience, Europe) at an appropriate wavelength (405nm, 633nm, and 488nm) and analyzed with FlowJo software.

### Conclusion

Using this protocol, we could stain immune cells (CD45), determine living vs. apoptotic cells, Treg cells (CD25), M1- (CD80), and M2- (CD163) macrophages obtained from the human omentum.

## Abstract BS-09

**CHARACTERIZATION OF HUMAN OMENTAL MILKY SPOTS BY IMMUNOHISTOCHEMISTRY**

Authors: Caroline Graf1, Frank-Jürgen Weinreich1, A. Königsrainer1,2, G. Nadiradze1,3, Marc A. Reymond1,3, and Wiebke Solass1,3

Institution: 1 National Center for Pleura and Peritoneum, NCT South-West Germany, 2 Dept. of General and Transplant Surgery, 3 Institute of Pathology and Neuropathology, University Hospital, Tübingen, Germany

### Background

MSs are unique lymphatic structures mainly located in the greater omentum and are the first line of defense in the early phase of PM. MSs also provide a structural and metabolic basis for PM’s development. The aim of this study was phenotyping of MS immune cells in the human omentum in patients with PM/without PM.

### Methods

Fixed human greater omentum samples were immersed in 1l distilled water containing 1g hematoxylin, 50g aluminum ammonium sulfate, 0.2g sodium iodide, 1g citric acid, and 50g chloral hydrate for 7 min. Probes were rinsed for 10 min in water. Stained MSs were localized with a stereomicroscope and placed in PBS/sucrose buffer. Samples were dehydrated in ethanol series of increasing concentration. After clearing in xylene, samples were embedded in paraffin, microtomed into 5 μm sections and mounted. After drying, sections were selected by microscopy for the presence of MS. Selected slides were stained using automatized staining protocols.

### Results

CD3, CD20, CD25, CD68, CD80, and C163- positive immune cell subpopulations were identified by IHC using the sample preparation technique above.

### Conclusion

To our knowledge, this is the first demonstration of MS immune cell subpopulations by immunohistochemistry in human omental samples. Thus, this protocol opens a new observational window for characterizing the peritoneal immunological space in health and disease.

## Abstract BS-10

**THE ROLE OF CYTOLOGY IN PATIENTS WITH PERITONEAL METASTASIS UNDER PRESSURIZED INTRAPERITONEAL AEROSOL CHEMOTHERAPY (PIPAC) TREATMENT**

Authors: Hugo Teixeira Farinha, Julien Châtelain, Daniel Clerc, Christine Sempoux, Nicolas Demartines, and Martin Hubner

Institution: Department of Visceral Surgery, Department of Pathology, Lausanne University Hospital (CHUV), University of Lausanne (UNIL), Switzerland

### Background

Cytology of ascites or peritoneal washing is routine part of intraoperative staging of peritoneal metastases. The aim of this study was to determine the added value of cytology in patients undergoing PIPAC.

### Methods

Retrospective monocentric cohort study of consecutive patients undergoing PIPAC including cytology assessment from January 2015 to January 2020.

### Results

A total of 78 patients were analyzed (median age: 65 years (SD 53-69)) including 144 PIPAC: 49% digestive, 35% ovarian, and 16% mesothelioma. Median PCI at 1st PIPAC was 14 (range:0-39). Patients had a median of 2 (1-2) lines and 6 (6-12) cycles of prior systemic chemotherapy. Overall cytology was positive in 59% of patients at 1st PIPAC, 75% in presence of ascites, and 44% for peritoneal washing. In patients having the scheduled 3 PIPAC, positive ascites was present in 65% and 70% at PIPAC1 and 3, respectively. 1 cytology+ patient became cytology– under PIPAC, and the opposite occurred in 2 patients. For cytology+ median PCI and mean ascites (ml) were: 9(3-19) vs 19(12-25) p=0.01 and 230ml (±100) vs 840ml (±150) p=0.01. Median PRGS was 1.2(1-1.9) in cytology– vs 2 (1.8-2.5) in cytology+ p=0.01. No difference was observed between cytology+ or – regarding disease progression (88% vs. 68%, p=0.10) and OS (50% vs. 65%, p=0.24) at 24 months. Negative cytology at PIPAC 3 was associated with 25% ascites, better PRGS but not with disease progression and survival. No treatment decisions were adapted based on cytology in our cohort.

## Abstract BS-11

**COMPARISON OF STAGING LAPAROSCOPY FOR PERITONEAL METASTASES WITH OR WITHOUT PRESSURIZED INTRA-PERITONEAL AEROSOL CHEMOTHERAPY (PIPAC)**

Authors: Daphné Mattille, Hugo Teixeira Farinha, Styliani Mantziari, Nicolas Demartines, Martin Hübner

Institution: Department of Visceral Surgery, Lausanne University Hospital (CHUV), University of Lausanne (UNIL), Switzerland

### Background

PIPAC has been introduced for palliative treatment of PM and is currently tested also in the neoadjuvant and prophylactic setting. The aim was therefore to compare safety and tolerance of staging laparoscopy with or without PIPAC.

### Methods

This retrospective analysis compared consecutive patients undergoing staging laparoscopy alone for oesogastric cancer with patients having PIPAC for suspected PM of various origins from January 2015 until January 2020. Safety was assessed by use of Clavien classification for complications and CTCAE for capturing of adverse events. Nausea was documented and pain by use of a visual analog scale (VAS:0-10: maximal intensity).

### Results

Overall, 25 PIPAC patients were compared to 24 in the laparoscopy group. 84% of PIPACs were performed with 2 trocars, while most laparoscopies (50%) required 3 trocars. PIPAC procedures took a median of 35 min (IQR: 25-67) longer. Four patients experienced at least one complication in either group 4 in PIPAC and 4 laparoscopy, respectively (p=0.741). No differences were noted for postoperative nausea (13 vs 8%, p=0.96) and pain levels (median (IQR) 1(0-2) vs 1 (0-2), p=0.16) between PIPAC and laparoscopy, respectively. Median hospital stay was 2 (IQR: 1-3) for PIPAC and 1 (IQR: 1-2) for laparoscopy (p=0.11).

### Conclusion

The addition of PIPAC did not jeopardize safety and tolerance of staging laparoscopy alone. Further studies need to clarify its oncological benefits.

## Abstract BS-12

**CISPLATIN UPTAKE IN OVARIAN CANCER PERITONEAL METASTASES AFTER HIPEC**

Authors: Jesse Demuytere1, Charlotte Carlier1, Tom Van Helden2, Joke Belza2, Frank van Haecke2, Joseph Weerts3, Sarah Cosyns1, and Wim Ceelen1,4

Institution: (1) Department of GI Surgery, UZ Ghent

(2) Department of Chemistry, Atomic and Mass Spectrometry, UGent

(3) Department of Surgery, CHC Liège

(4) Cancer Research Institute Ghent (CRIG)

### Background

The addition of HIPEC to cytoreductive surgery in stage III epithelial ovarian cancer improves outcomes. However, the impact of hyperthermia on pharmaco-kinetics and -dynamics *in vivo* remains to be defined. Here, we report *in vivo* tumor uptake of cisplatin of patients enrolled in the OvIP study (NCT02567253), a multicenter randomized trial investigating tumor tissue platinum penetration after normothermic (37°C) versus hyperthermic (41°C) chemoperfusion with cisplatin at 75 or 120 mg/m2.

### Methods

Following cisplatin HIPEC of 90 minutes, we obtained tumor samples in each patient (n=54). After microscopic selection of eligible samples, the platinum distribution in 21 samples was analyzed using LA-ICP-MS. Tissue regions and histology were annotated on consecutive HE sections.

### Results

Platinum distribution in tumor tissues was heterogeneous, with a clear gradient of uptake with high levels of intensity at the treated peritoneal membrane diminishing towards deeper layers of the submesothelial stroma. Platinum signal in malignant cells was lower than the surrounding tumoral stroma, with a mean ratio of platinum signal intensity between stromal and cancer cells of 2,4 (SD 0,887). No statistically significant differences were observed when comparing for treatment dose level or temperature.

### Conclusion

HIPEC with cisplatin for 90 minutes leads to limited uptake in neoplastic cells, compared to their microenvironment.

## Abstract BS-13

**RESISTANCE TO INTRAPERITONEAL DRUG DELIVERY AND HETEROGENEITY OF PERITONEAL METASTASIS: THE ROLE OF HYDRAULIC CONDUCTIVITY**

Authors: Hooman Salavati (1,2,3), Charlotte Debbaut (2,3), Pim Pullens (4,5), and Wim Ceelen (1,3)

Institution: (1) Department of Human Structure and Repair, Ghent University, Ghent, Belgium

(2) IBitech– Biommeda, Ghent University, Ghent, Belgium

(3) Cancer Research Institute Ghent (CRIG), Ghent, Belgium

(4) Department of Radiology, University Hospital Ghent, Ghent, Belgium

(5) Ghent Institute of Functional and Metabolic Imaging (GIFMI), Ghent University, Ghent, Belgium

### Background

Ip drug delivery to solid tumors is hindered due to a set of biophysical barriers including elevated interstitial fluid pressures (IFP). Although the tumor hydraulic conductivity (K) has been found to strongly correlate with IFP, the availability of K values in human cancer tissue is very limited. Here, we present a novel *in vitro* setup to measure K in clinical samples.

### Methods

An apparatus was developed based on modified using diffusion chambers. The setup comprises the chambers along with a bubble tracker device for quantifying the amount of fluid exchange through the tissue due to a hydrostatic pressure gradient in a closed system. A 3D realistic geometry of a peritoneal tumor was used in a computational fluid dynamics (CFD) study to simulate the sensitivity of IFP profiles to K values.

### Results

The measured values (K ranging between 5.1E-15 and 4.1E-14 m2/Pa∙s) demonstrated good agreement with previously published values, which were either measured in animal studies or estimated indirectly. The results indicated heterogeneity of hydraulic conductivity in a single tumor up to a factor 2.7. Moreover, K values in the same tumor type varied significantly. The CFD model showed that changes in K may remarkably affect the IFP.

### Conclusion

We successfully built a setup that allows to measure hydraulic conductivity of human cancer tissue samples. The results can inform novel ip delivery approaches that target the biomechanical tumor environment.

## COLORECTAL CANCER PERITONEAL METASTASES

**Invited lecture**

**PROGNOSTIC VALUE OF MUTATIONAL STATUS IN CRC PERITONEAL METASTASES. ITALIAN ONCOTEAM EXPERIENCE**

Authors: Antonio Sommariva, Marco Tonello, Dario Baratti, Paolo Sammartino, Andrea Di Giorgio, Manuela Robella, Cinzia Sassaroli, Massimo Framarini, Mario Valle, Antonio Macrì, Luigina Graziosi, Federico Coccolini, Piero Vincenzo Lippolis, Gelmini Roberta, and Marcello Deraco

Institution: Veneto Institute of Oncology IOV-IRCCS Padova Italy

Content: Selection of patients with CRC PM which might benefit from CRS and HIPEC remains crucial. Main aim of this multicenter study was to analyze prognostic impact of RAS/RAF mutations and microstallite (MS) status in patients with CRC PM treated with CRS-HIPEC in 13 Italian peritoneal cancer centers. Between 2003–2019, 437 patients treated with cytoreductive surgery and HIPEC have been collected. After a median follow-up of 37.7 months (95% CI 34.4-48.8), OS was 42.3 months (95% CI 33.4-51.2), DFS 13.6 months (95% CI 12.3-14.9) and local-DFS (peritoneal only) 20.5 months (95% CI 16.4-24.6). At multivariable analysis, among known clinical and pathological factors (PCI, residual disease, nodal status, signet ring histology), KRAS mutation (HR 2.0, 95%CI 1.3-2.9, p 0.0001) and BRAF mutation (HR 3.3, 95%CI 1.7-6.1, p 0.002) were related to OS, whereas MSI was not (p 0.68). Developing a multivariable model with a combination variable including KRAS/BRAF mutation and MS, MSI/WT patients (HR 0.4 95%CI 0.1-1.1, p 0.08) and MSI/mutated or MSS/WT (HR 0.5 95%CI 0.3-0.7, p 0.0001) have an improved survival. In conclusion, RAS/RAF mutations and MS status should be strongly considered in the selection process of patients potentially eligible for cytoreductive surgery, as MSI confers a significant survival advantage over stable patients also in RAS/RAF mutated patients.

## Abstract CRC-01

**PROGNOSTIC IMPACT OF SIGNET RING CELL PROPORTION IN COLORECTAL CANCER PATIENTS WITH PERITONEAL METASTASES**

Authors: Vahan Kepenekian, Amaniel Kefleyesus, George Petrides, Shoma Barat, Isabelle Bonnefoy, Sarah Valle, Olivier Glehen, and David L. Morris

Institution: Lyon University Hospital, Lyon-Sud, France

### Background

Rarely, CRCs comprise signet ring cells (SRC) made of cytoplasmic mucin and responsible for a poor prognosis. The prognostic impact of the SRC component (SRCc) in CRC with peritoneal metastases (pmCRC) candidate for cytoreductive surgery remains unclear, jeopardizing patients selection.

### Methods

A retrospective analysis of a bicentric cohort was performed to assess the impact of SRC proportion by comparison to classical adenocarcinoma (cADK) regarding oncologic outcomes. The SRC proportion was reviewed by an expert pathologist.

### Results

Overall, 59 pmCRC patients with SRC were included (15 with SRCc >50%) and compared to 647 cADK patients with peritoneal metastases. When compared to cADK, the SRC patients were younger, more often with right-sided tumors, with pN2 status and with higher peritoneal metastasis index (median 16.0 vs 8.6, respectively). After a median follow-up of 40 months [IC 95%, 36-45], the median overall survival was 14, 20 and 42 months in patients with ≤50% SRC, >50% SRC and cADK respectively (p<0.001). In multivariate analysis SRCc ≤50% (HR 2.85, [IC95%, 1.28-6.35], p<0.001) and SRCc >50% (HR 7.75, [IC95%, 3.58-16.75], p<0.001) were associated with a poorer survival, while perioperative and adjuvant chemotherapy (HR 0.27, p=0.033; HR 0.22, p=0.003) led to a survival advantage.

### Conclusion

In pmCRC patients candidate for cytoreduction, any component of SRC was an independent bad prognosis factor: the higher the component was, the poorer was the prognosis.

## Abstract CRC-02

**PREDICTING SURGICAL FAILURE IN MALIGNANT BOWEL OBSTRUCTION SECONDARY TO PERITONEAL METASTASIS**

Authors: Claudio Lodoli1*, Marcello Covino2,5*, Miriam Attalla El Halabieh1,5, Francesco Santullo1, Andrea Di Giorgio1,5, Carlo Abatini1,5, Stefano Rotolo1, Federica Ferracci1,5, Elena Rodolfino4,5, Anna Fagotti3,5, Giovanni Scambia3,5, Francesco Franceschi2,5, and Fabio Pacelli1,5.

Institution: 1 Surgical Unit of Peritoneum and Retroperitoneum Surgery, Fondazione Policlinico Universitario A. Gemelli, IRCCS 2 Emergency Medicine, Fondazione Policlinico Universitario A. Gemelli, IRCCS. 3Division of Gynecologic Oncology, Department of Women and Children’s Health, Fondazione Policlinico Universitario A. Gemelli, IRCCS 4 Department of Radiology, Fondazione Policlinico Universitario A. Gemelli, IRCCS. 5Università Cattolica del Sacro Cuore, Rome, Italy

*Share first co-authorship.

### Background

Malignant bowel obstruction (MBO) secondary to PM is a common evolution in patients with end-stage abdominal malignancies and is associated with a poor prognosis. The role of palliative surgery remains unclear. The aim of this study is to identify criteria to select patients who could benefit from invasive surgical interventions, and in which cases medical palliation would be the preferred strategy.

### Methods

In this study 98 consecutive patients who underwent palliative surgery for MBO over a period of 5 years were reviewed retrospectively. Outcome measures were surgical failure considered as any ineffective laparotomy and successful palliation following surgery defined as the ability to resume oral diet at the time of discharge from the hospital. Based on a logistic regression model, a prognostic score was developed to identify patients at risk of surgical failure.

### Results

Successful palliative surgery was achieved in 76 (77.5%) patients. Level of bowel dilatation (p=0.046) and site of bowel obstruction (p=0.012) emerged as independent predictors of surgical failure. Metastasis level Assessment for peritoneum–CLAP score based on these factors was built to evaluate the risk of surgical failure.

### Conclusion

This scoring system might help select patients most likely to benefit from palliative surgery.

## Abstract CRC-03

**A MODEL TO REFINE THE IDEAL TIMING FOR LAPAROSCOPIC EXPLORATION OF PERITONEAL METASTASIS IN COLONIC CANCER**

Authors: Fawaz Jade, Hobeika Christian, Liberal Gabriel, Eveno Clarisse, Malgras Brice, Sideris Lucas, Hubner Martin, Sabbagh Charles, Sgarbura Olivia, Taibi Abdel, and Pocard Marc

Institution: Hôpital La Pitié Salpêtrière

### Background

Laparoscopic exploration (LE) is an asset in colonic PM, provided that it is performed neither early nor late. This study aimed to refine the ideal timing for LE.

### Methods

This international cohort-study included patients with metachronous PM of operated colonic adenocarcinoma between 2001–2021. Patients with synchronous PM (PCI>4), positive margins or pNx were excluded. Cox regression was used to assess factors associated with PM-free survival (PMFS). Survival tree and predictive model were fitted and validated.

### Results

Of 235 patients included, 46% were men. Median age was 58 years (IQR:49-64). Median PMFS was 13 months (IQR:12-16). pT4 (HR=1.47;95%CI:1.03-2.1), pN1 (HR=0.58;95%CI:0.37-0.92), synchronous PM (HR=2.71,95%CI:1.76-4.16), transverse localization (HR=0.35;95%CI:0.18-0.69), surgery in emergency (HR=2.19,95%CI:1.48-3.22), MSI (HR=2.29;95%CI:1.04-5.03), and KRAS-mutated (HR=1.78;95%CI:1.24-2.55) were independently associated with PMFS. Based on a survival tree, PMFS varied from 3.6 to 20.6 months according to these factors. A model was created (AUC:0.71; optimim2:0.02) to predict the probability to have PM at a given time point of the follow-up. A cut-off to exclude PM relapse was identified (accuracy:86.4%; positive predictive value:95.4%).

### Conclusion

The model helps to refine, on a case-by-case basis, the optimal indication, and timing for LE, going from a systematic second look to a delayed LE according to prognostic factors.

## Abstract CRC-04

**THE IMPACT OF HIGH INTRA-ABDOMINAL PRESSURE DURING HYPERTHERMIC INTRAPERITONEAL CHEMOTHERAPY ON CLINICAL OUTCOMES IN PATIENTS FOLLOWING CYTOREDUCTIVE SURGERY FOR PERITONEAL SURFACE-BASED MALIGNANCIES**

Authors: Louis Choon Kit Wong*, Jolene Si Min Wong*, Chin-Ann Johnny Ong, and Claramae Shulyn Chia

* equal contribution

Institution: National Cancer Center Singapore

### Background

CRS and HIPEC play key roles in the treatment of peritoneal malignancies. There is evidence that HIPEC at high intraabdominal pressure (IAP) results in increased tissue penetration. We thus aim to evaluate differences in perioperative outcomes in patients undergoing CRS-HIPEC with low versus high IAP.

### Methods

Patients undergoing CRS-HIPEC were recruited from Jan 2020 to Feb 2021 and stratified into 2 groups based on IAP, with low IAP defined as <18mmHg and high IAP as ≥18mmHg. Intra- and post-operative events were recorded. Comparisons were made with independent and paired-samples t-tests, and linear mixed models for groups of repeated measures.

### Results

A total of 33 patients underwent CRS-HIPEC (n low=13, n high=20). Mean IAP in the low and high IAP groups were 13.1 and 19.7mmHg, respectively. When comparing physiological changes during HIPEC, there were no significant differences between the 2 groups. Both experienced increase in heart rate (MD=12.6±8.1, p<0.01) and central venous pressure (MD=4.1mmHg±3.2, p<0.01) with decrease in mean arterial pressure (MD=4.8mmHg±8.2, p<0.01). There was also similar increase in base deficit (MD=3.4mmol/L±2.5, p<0.01) and serum glucose (MD=3.3mmol/L±1.3, p<0.01). Postoperatively, high IAP did not result in increased rate of complications, time to full feeds, ICU or total hospital stay.

### Conclusion

High IAP in HIPEC is safe and did not result in additional adverse events.

## Abstract CRC-05

**ASSESSMENT OF TREATMENT RESPONSE AFTER PRESSURIZED INTRAPERITONEAL AEROSOL CHEMOTHERAPY (PIPAC) FOR APPENDICULAR PERITONEAL METASTASES**

Authors: SP Somashekhar1*, Julio Abba2*, Olivia Sgarbura3, Mohammad Alyami4, Hugo Teixeira Farinha5, Ramya G Rao1, Wouter Willaert6, and Martin Hübner5 on behalf of the PIPAC study group

*Equal contribution

Institution: 1 Department of Surgical Oncology, Manipal Comprehensive Cancer Center, Manipal Hospital, Bengaluru India. 2 University Hospital Grenoble, France. 3 Department of Surgical Oncology, Cancer Institute Montpellier (ICM), Montpellier, France; University of Montpellier, France. 4 Department of General Surgery and Surgical Oncology, Oncology Center, King Khalid Hospital, Najran, Saudi Arabia. 5 Department of Visceral Surgery, University Hospital CHUV and University of Lausanne(UNIL), Lausanne, Switzerland 6 Department of human structure and repair, Ghent University, Ghent, Belgium

### Background

PIPAC is a new treatment with promising safety profile and early response rates for PM of various origins. We aimed to analyze oncological outcomes after PIPAC for appendicular tumors.

### Methods

This retrospective cohort study included consecutive patients with appendicular PM treated in experienced PIPAC centers (>60 procedures). The primary outcome measure was OS. Secondary outcome measures included radiological response (RECIST criteria), PCI, histological response assessed by the peritoneal regression grading system (PRGS: complete response: 1 - 4: no response), and symptoms.

### Results

A total of 77 consecutive patients were included (208 PIPAC procedures) from 15 centers. Median OS was 20.9 months (IQR13.7-31.4) from time of diagnosis and 9.9 months (IQR 4.5-20.8) from start of PIPAC treatment. A total of 35/77 patients (45%) had ≥3 procedures (pp: per protocol); objective response at PIPAC3 was as follows: RECIST: partial response 6 (7.8%), 18 (23.4%) stable; mean PRGS: 1.8±0.9 vs. 2.5±1.3 (baseline, p=0.0002); median PCI: 21 (IQR18-27) vs. 22 (IQR17-28) at baseline (p=0.591); 20 (58.8%) and 17 (50.0%) patients were symptomatic at baseline and PIPAC 3, respectively (p=0.465). Median OS (pp cohort) was 16months (IQR7.3-26.2) from 1st PIPAC.

### Conclusion

Patients with PM of appendicular origin have objective treatment response after PIPAC and encouraging survival curves call for further prospective evaluation.

## Abstract CRC-06

**TREATMENT RESPONSE AFTER PRESSURIZED INTRAPERITONEAL AEROSOL CHEMOTHERAPY (PIPAC) FOR PERITONEAL METASTASES OF COLORECTAL ORIGIN**

Authors: Martin Hübner1*, SP Somashekhar2*, Hugo Teixeira Farinha1, Julio Abba3, Ramya G Rao2, and Wouter Willaert4 on behalf of the PIPAC study group

*equal contribution

Institution: 1 Department of Visceral Surgery, University Hospital CHUV and University of Lausanne (UNIL), Lausanne, Switzerland 2 Department of Surgical Oncology, Manipal Comprehensive Cancer Center, Manipal Hospital, Bengaluru, India. 3 University Hospital Grenoble, France. 4 Department of human structure and repair, Ghent University, Ghent, Belgium

### Background

PIPAC is safe and well-tolerated for patients with PM. Our aim was to report oncological outcomes after PIPAC for colorectal PM.

### Methods

International retrospective cohort study of consecutive patients with colorectal PM. Outcome were OS, radiological response (RECIST), histological response by use of PRGS and cytology, PCI and symptoms.

### Results

A total of 17 eligible centers compiled 256 nonselected patients (mean age 59±13-year, 43% female) and 606 PIPACs. 55.4% were treated after 2 lines of prior chemotherapy and median PCI at 1st PIPAC was 18 (IQR10-27). Median OS was 19 months (IQR12.9-29.8) from diagnosis and 9.4 months (IQR4.5-16.8) from 1st PIPAC. A total of 104/256 patients had ≥3PIPACs (pp: per protocol) with following outcomes: RECIST: A total of 42 (16.4%) partial remission, 52 (20.3%) stable; mean PRGS: 2.6±0.8 (baseline) vs. 2.1± 0.9 at PIPAC3 (p=0.001); negative cytology at PIPAC3 in 34 patients (33%); median PCI was 21 (IQR15-29) at baseline and 20 (IQR12-27) at PIPAC3 (p=0.02). A total of 49 (49.0%) and 57 (55%) patients were symptomatic at baseline and PIPAC3, respectively (p≤0.08). Median OS for the pp cohort was 11 months (IQR7.1-17.7) from 1st PIPAC. Predictors for survival were radiological response HR3.0 (95% CI1.6-5.7) and no symptoms HR4.5 (95% CI2.2-9.1) at PIPAC3.

### Conclusion

Objective treatment response was demonstrated after PIPAC for colorectal PM, and preliminary survival curves are calling for prospective validation.

## Abstract CRC-07

**FECAL DIVERSION IN ANTERIOR RECTAL RESECTION AND ANASTOMOSIS DURING CYTOREDUCTIVE SURGERY AND HYPERTHERMIC INTRAPERITONEAL CHEMOTHERAPY FOR PERITONEAL METASTASIS: IS IT ALWAYS NECESSARY?**

Authors: Dr. Erion Rreka, Dr. Lorenzo Piccini, Dr. Mauro Ferrari, Dr.ssa Barbara Musco, Luca Tessieri, and Dr. Piero Vincenzo Lippolis

Institution: SC Multidisciplinare Centro Clinico Chirurgia del Peritoneo- SD Chirurgia Generale e Peritoneale Azienda Ospedaliero Universitaria Pisana

### Background

Extraperitoneal rectal resection in CRS/HIPEC for peritoneal metastasis is frequently associated with diverting loop ostomy to reduce the clinical impact of anastomotic leak.

### Materials and Methods

We retrospectively analyzed 184 CRS/HIPEC procedures for peritoneal metastasis between January 2016 and June 2021 at General and Peritoneal Surgery. We evaluated all patients who underwent rectal resection with Knight–Griffen pelvic anastomosis (colorectal, ileorectal). When possible a second layer of continuous suture was placed circumferentially to reinforce the stapled anastomoses.

### Results

Among 184 CRS/HIPEC patients 64 (34.8%) underwent rectal resection. 58 (90.6%) had pelvic anastomosis, 57 (89.1%) without ostomy, 1 (1.6%) with protective ileostomy. 6 (9.4%) patients had no anastomosis [4(6.2%) with end ileostomy, 2 (3.1%) with end colostomy]. The median age was 57, 17 (26.5%) were male and the median peritoneal cancer index was 13.8. Of 58 pelvic anastomosis patients, 3 (5.2%) had rectal anastomotic leak. A total of 4 (6.3%) patients had Clavien–Dindo IIIB complications and 6 (9.4%) IIIA complications. Mortality at 30 and 100 days was 0% and 4.6%, respectively.

### Conclusion

During CRS/HIPEC, pelvic bowel reconstruction associated with a second layer of circumferential suture without protective ostomy is safe. Fecal diversion in pelvic anastomoses should be performed in selected patients and should not be the rule.

## Abstract CRC-08

**INFLUENCE OF OTHER METASTATIC SITES ON THE CURATIVE MANAGEMENT OF PERITONEAL COLORECTAL METASTASIS**

Authors: F. Schell(a), V. Kepenekian(a, b), N. Benzerjeb (c), M. Chauvenet (d), E. Cotte(a, b), G. Passot (a,b), D. Vaudoyer (a,b), J. Perron(e), and O. Glehen (a,b)

Institution: (a) Department of Digestive and Oncologic Surgery, Hospices Civils de Lyon, Center Hospitalier Lyon Sud Pierre Bénite, Lyon, France, (b) Equipe CICLY, Université Lyon 1, Lyon, France, (c) Department of Pathology, Hospices Civils de Lyon, Center Hospitalier Lyon Sud Pierre Bénite, Lyon, France; (d) Department of Gastro-Enterology, Hospices Civils de Lyon, Center Hospitalier Lyon Sud Pierre Bénite, Lyon, France; (e) Department of Oncology, Hospices Civils de Lyon, Center Hospitalier Lyon Sud Pierre Bénite, Lyon, France

### Background

The prognosis of patients presenting with peritoneal metastasis from colorectal cancer (PMCRC), associated with extraperitoneal disease (EPD) treated surgically, remains a therapeutic challenge. Aim was to determine whether extraperitoneal metastatic sites (EPMSs) influence the prognosis of patients with PMCRC, when treated with a curative intent.

### Methods

We included patients with PMCRC undergoing CRS and HIPEC from 2005 to 2018. We identified patients with or without EPD to compare survival depending on the number of EPMS: Peritoneal disease only (PDO), one EPMS (1EPMS), and two or more EPMS (2+EPMS). Then, we analyzed survival depending on the location of the EPMS.

### Results

We identified 455 patients undergoing CRS+HIPEC for PMCRC, 143 had EPD. One hundred and twelve had 1 EPMS and 31 had 2+EPMS. Locations of EPD were: Liver metastasis (N=104), lung metastasis (N=19), and retroperitoneal lymph node (RLN) invasion (N=30). There was no significant difference in overall survival between PDO and 1EPMS groups, (64.6 and 52.2 months, respectively), but was poorer in the 2+EPMS group (29.4 months, p=0.002). Hepatic and lung metastasis did not impact survival (p=0.481 and p=0.751, respectively), whereas RLN invasion did (p=0.001).

### Conclusion

Limited EPD (liver and or lung metastasis) in patients with PMCRC treated curatively did not influence survival whereas RLN and presence of two metastatic sites decrease survival.

## Abstract CRC-09

**EVALUATION OF NEOADJUVANT CHEMOTHERAPY FOR THE CURATIVE MANAGEMENT OF PERITONEAL METASTASIS FROM COLORECTAL CANCER ON MORPHOLOGICAL AND PATHOLOGICAL RESPONSES**

Authors: Vahan Kepenekian (a,b), Julien Peron (c), Isabelle Bonnefoy (a,b), Mohammad Alyami (d), Laurent Villeneuve (b), Naoual Bakrin (a,b), Pascal Rousset (b,e), Benoit You (b,c), Nazim Benzerdjeb (b,f), and Olivier Glehen (a,b)

Institution: (a) Department of Oncologic and General Surgery, Hospices Civils de Lyon, Center Hospitalier Lyon Sud Pierre Bénite, (b) Equipe CICLY, Université Lyon 1, (c) Department of Medical Oncology, Hospices Civils de Lyon, Center Hospitalier Lyon Sud Pierre Bénite, (d) Department of Digestive Surgery, King Khalid Hospital, Najran, Saudi Arabia (e) Department of Radiology, Hospices Civils de Lyon, Center Hospitalier Lyon Sud Pierre Bénite, (f) Department of Pathology, Hospices Civils de Lyon, Center Hospitalier Lyon Sud Pierre Bénite, Lyon, France

### Background

There is currently no evidence of efficacy of neoadjuvant chemotherapy (NC) in the curative management of PMCC treated by CRS alone or combined with HIPEC. Aim was to assess the morphological and pathological tumor responses rates (MR, PR) according to various NC regimens.

### Methods

Patients who underwent a preoperative chemotherapy followed by complete CRS alone or combined with HIPEC between January 2005 and June 2018 were retrospectively included. MR was assessed by comparison of radiological imaging. PR was defined as the mean percentage remaining of tumor in surgical specimen. Univariate and multivariate analysis were performed to identify predictors of survival, MR and PR.

### Results

A total of 440 patients were included. MR rates were 44/137 patients (44.0%) for doublet chemotherapies alone, 146/243 (65.2%) when combined with targeted therapies and 39/60 (65.0%) for triplet chemotherapy (p=0.002). A total of 31/137 (41.9%) patients achieved a complete or major PR with bichemotherapy alone, 66/243 (43.8%) when combined with targeted therapies and 15/60 (41.7%) in the group Folfirinox (p=0.064). On multivariate analysis, NC regimens were not a predictive factor of 3-years overall or disease-free-survival.

### Conclusion

NC combined with targeted therapies and triplet chemotherapy regimens may lead to better MR but did not significantly increase the PR of PMCC without influence on overall survival.

## Abstract CRC-10

**INTRAOPERATIVE ADJUVANT HYPERTHERMIC INTRAPERITONEAL CHEMOTHERAPY IN PATIENTS WITH LOCALLY ADVANCED COLON CANCER: A PROSPECTIVE PARALLEL PHASE II STUDY**

Authors: Macaione Ina, Mandalà Lucio, Campisi Antonella, Roz Elena, Mercadante Sebastiano, Piazza Dario, Cipolla Calogero, Mezzatesta Pietro, and Gebbia Vittorio

Institution: La Maddalena, Palermo, Sicily, Italy

### Background

Prophylactic HIPEC showed promising Results in patients with colorectal carcinoma at high risk of recurrence but still without clinically and radiologically evident signs of peritoneal spread. This study aims to analyze the feasibility and safety of this proactive, early phase, multimodality approach in terms of length of hospital stay, surgical, and medical treatment-related toxicity.

### Methods

A mono-institutional, prospective, parallel, two-stage phase II trial enrolled 49 patients to standard surgery or surgery plus intraoperative HIPEC. Before the procedure and during surgery, patients received intravenous fluorouracil (and leucovorin to potentiate OXA activity. Data analysis included length of hospital stay, surgery duration, type of surgery, and chemotherapy-related complications risk score.

### Results

No significant difference was seen in the median time spent in the hospital with a median stay of 7 days in both groups (p=0.5720). The surgical procedure’s median duration was longer in the HIPEC group than in the control one. Side-effects and surgical complications did not cross at any time the Pocock-type boundary for side/effect monitoring (p=0.80, N.S.).

### Conclusion

The present prospective study results demonstrate the feasibility and safety of the colorectal surgery plus HIPEC treatment in patients with colorectal cancer patients at high-risk for peritoneal invasion, although clinically and radiological -TRUNCATED AT 1500 CHARACTERS

## Abstract CRC-11

**EFFECT OF SURGICAL HUMIDIFICATION ON LOCAL AND SYSTEMIC INFLAMMATION AND PERITONEAL TRAUMA IN COLORECTAL CANCER SURGERY: A RANDOMIZED CONTROLLED TRIAL**

Authors: R Ramsay1, S Sampurno1, M Dean1,T Chittleborough1, S Carpinteri1, R Millen1, J Hiller, S Roth1, S Warrier1, A Heriot1, and A.C Lynch2

Institution: Peter MacCallum Cancer Center and the Epworth Hospital

### Background

Carbon dioxide(CO_2_)-mediated insufflation in laparoscopic surgery in patients with colorectal cancer was investigated.

### Methods

Sixty-nine patients with were randomized; those being insufflated with drycold- CO_2_ (DC-CO_2_) or humidified warm-CO_2_ (HW-CO_2_). A total of 19 patients undergoing laparotomy were randomized to conventional surgery or with a perfusion device into the abdominal cavity delivering HW-CO_2_. Core temperatures were monitored presurgery and during the surgery. Peritoneal biopsies and blood were taken at the start of surgery, at 1, 3, and up to 6 hours.

### Results

Most patients in the laparoscopic arms experienced a temperature drop despite uniform use of Bair HuggerTM. HW-CO_2_ restored to, and maintained normothermia (≥ 36.5oC) by 3 hours, DC-CO_2_ did not. On average patients in both laparotomy arms were colder and failed to return to normothermia while in the operating theater. Peritoneal samples were collected and subjected to patient-blinded evaluation using scanning electron microscopy (SEM) to evaluate mesothelial cell and microvilli damage. Unexpectedly approximately one third of patients across all arms had preexisting peritoneal damage. Subsequent to surgery peritoneal damage increased at 1 and 3 hours to a greater extent in the DC-CO_2_ compared to the HW-CO_2_ laparoscopic cohort. Inflammatory markers evaluated over 0-5 days, were consistently higher in open surgery cases than laparoscopic cases and lower in matched groups that employed HW-CO_2_.

### Conclusion

This RCT confirms animal model observations.

## Abstract CRC-12

**ORGANOID BASED PERSONALIZED MEDICINE FOR PATIENTS WITH COLORECTAL PERITONEAL METASTASES: CAN IT BE DONE?**

Authors: V Narasimhan 1, M Flood 1, J Wright 2, M Churchill 3, M Michael 1, C Grandori 3, J. Tie 1, D Worthley 2, S Woods 2, A Heriot 1, and R Ramsay 1

Institution: 1 Peter MacCallum Cancer Center, Melbourne, Australia, 2 South Australia Health and Medical Research Institute, Adelaide, Australia, 3 SEngine Precision Medicine, Seattle, USA

### Background

The majority of patients with CRPM are inoperable, rendering systemic chemotherapy as the mainstay of treatment. However, chemotherapy has poor efficacy, with treatment failures common. Patients are treated with generic chemotherapy regimes without any knowledge of each tumors sensitivity to a prescribed drug. Advances in preclinical modeling of disease in the form of organoids have led to real-time functional assessment of a tumor’s sensitivity to a specific drug. Here, we aim to establish and evaluate the feasibility of a novel organoid-based platform to integrate genomic and functional drug sensitivities to deliver personalized therapy to patients with treatment-refractory CRPM.

### Methods

Operative biopsies from CRPM patients were used to grow organoids. Organoids were validated and sequenced for mutational aberrations, before undergoing throughput drug testing with over 50 FDA approved drugs.

### Results

Organoids were established and screened against a combination of chemotherapeutics. Drug sensitivity in-vitro appeared to consistently mirror patient responses to the same drug in-vivo, confirming the validity of the platform. Novel therapies were detected in many patients with treatment refractory CRPM, when no genomic driven biomarkers were evident, underscoring the value of functional testing in providing diverse treatment options.

### Conclusion

An organoid-based personalized medicine platform is feasible with promising early Results.

## Abstract CRC-13

**IS THE BRAF MUTATION A CONTRAINDICATION TO A MULTIMODAL TREATMENT IN PATIENTS WITH PERITONEAL METASTASES FROM COLORECTAL CANCER? A RETROSPECTIVE PRELIMINARY TRIAL**

Authors: Jade Fawaz, MD; Andre Thierry, MD, PHD; Rachid KACI, MD; Brice MALGRAS, MD, PhD; Marc POCARD, MD, PhD; and Rea LO DICO, MD, PhD

Institution: Department of Digestive and Oncological Surgery, Lariboisière University Hospital, APHP, Paris, France

### Background

OS for patients with metastatic CRC and BRAF V600E mutation is limited. Surgery remains controversial. The aim of this study is to evaluate long-term outcomes of patients with PM from CRC and BRAF mutation treated by multimodal treatment (MMT) with a curative intent.

### Methods

Institutional database was used to select patients with BRAF mutation retrospectively. Patients received MMT: Cytoreductive surgery (CRS) plus HIPEC and perioperative chemotherapy. The progression free survival (PFS) was calculated from CRS + HIPEC to relapse and OS from the diagnosis of PM to the date of death or latest news.

### Results

Between 2009 and 2018, 24 patients with PM from CRC with BRAF V600E mutation were identified. MMT was realized for 15 patients mean aged of 58 years (IQR 34–69). Mean PCI was 9 (IQR 2–23). The postoperative morbidity rate was 30% (n=5); with no postoperative mortality. The mean follow-up was 24 (IQR 5-56) months. Median PFS and OS were 12 (IQR 1,6-30,1) and 28 (IQR 8,4-58) months, respectively. We observed 8 long survival patients with an OS longer than 24 months.

### Conclusion

This study showed encouraging survival after MMT. BRAF mutation is a negative prognostic factor but its association with PM does not represent a contraindication to MMT, since the prognosis is due to the presence of PM. Patient selection is necessary and further studies are required.

## Abstract CRC-14

**MALIGNANT BOWEL OBSTRUCTION RELATED TO ADVANCED PERITONEAL METASTASES: OUTCOMES OF SURGICAL TREATMENT**

Authors: Susanna Scarsi, Hugo Teixeira Farinha, Nicolas Demartines, Martin Hübner, and Daniel Clerc

Institution: Department of Visceral Surgery, Lausanne University Hospital (CHUV), University of Lausanne (UNIL), Lausanne, Switzerland

### Background

MBO related to advanced PM is related to poor survival. Surgical treatment is frequently the sole treatment option, but operative risks are high. The aim of this study was to assess the outcomes of patients operated for MBO related to PM.

### Methods

Single-center retrospective analysis of consecutive patients operated of refractory MBO of various origins from 2016 to 2021. Primary outcome was OS. Secondary outcomes were morbidity, resumption of chemotherapy, and reobstruction.

### Results

Twenty-nine patients (median 64 years, 59% female) were included. Median PCI was 31, and ascites was present in 48% of patients. Surgical resolution of obstruction was achieved in 79% of patients by resection (41%), internal bypass (17%), or stoma creation (17%). Major morbidity rate was 28% with no postoperative mortality. Four patients (14%) were reoperated and 3 patients (10%) developed enterocutaneous fistula. Median OS was 4.7 months. OS at 3, 6, and 12 months was 52%, 38%, and 24%, respectively. Chemotherapy was resumed in 20 patients (69%) and 12 (41%) developed reobstruction. Ascites was more frequently present in patients with <6 months survival (67%, p=0.01).

### Conclusion

Surgery is a valid option for patients presenting MBO related to advanced PM. Although morbidity is high, most patients can resume systemic chemotherapy postoperatively. Presence of ascites appears to be a negative predictor of survival.

## Abstract CRC-15

**INITIAL EXPERIENCE IN LAPAROSCOPIC PERITONECTOMY AND HIPEC FOR THE TREATMENT OF PERITONEAL METASTASIS IN A NEWLY ESTABLISH CENTER**

Authors: Samer Alammary, Abdullah AlQattan, Ahmad Elsaied, Rawaby Madkhali, Abdulbari Alewadhi, Mohammad Aljamahir, Mahdy Alzamanan, Mashhour AlQannas, Mohammad ALYami, and Delia Cortes Guiral

Institution: King Khalid Hospital, Najran, Saudi Arabia

### Background

Laparoscopic peritonectomy has been described as a feasible and reproducible technique, however it is technically challenging and requires specific training. Our aim is to present our experience on laparoscopic peritonectomy and laparoscopic HIPEC in a newly stablish center.

### Methods

A retrospective analysis from March 2019 to June 2021 from the King Khaled Hospital (Najran, Saudi Arabia) HIPEC data base has been conducted for every patient who underwent complete laparoscopic cytoreductive surgery with HIPEC. An exhaustive registry was made of all clinical relevant data, surgical procedure, outcome criteria as overall survival and adverse events according to (CTCAE) version4.0. All procedures were done by well-trained surgeons.

### Results

Excluding cases converted to open due to adhesions, 5 patients were analyzed, 3 of them were female. Median age was 43 years. PM was from appendix, colorectal, and ovarian cancer for 3, 1, and 1 patient, respectively. Median PCI was 8 (3–18) and median time of surgery (excluding HIPEC time) was 8 hours (4.5–10.5). Hospital median length of stay was 8 days (only one patient was admitted for 2 days in ICU).All patients underwent resection of the primary tumor, parietal peritonectomy and one of them total pelvic peritonectomy plus salpingo-oophorectomy with hYsterectomy and extraction of the specimen through the vagina, 2 patient underwent bowel resection with anastomosis. Three patients had complications, grade IV (hemoperito - TRUNCATED AT 1,500 CHARACTERS

## Abstract CRC-16

**FLUOZEROC TRIAL (FLUORESCENCE AS A TRACKER FOR A ZERO CC0 CYTO-REDUCTION FOR COLO-RECTAL PERITONEAL METASTASES)**

Authors: Abdulbari Alawadhi1, Samer Ali Al Ammari2, Muath Riyadh Ali2, Ahmed Hefny Elsaied2, Rawabi madkhli2, Mana Ali Mueidh Al Hajlan2, Mohammed Yahya Al- Jamahir2, Mahdy Yahya Al Zamanan1, Saad Mohammad Almowallad2, Isabel Prieto Nieto3, Mohammed Saleh ALyami2, Diana Michele4, Tahir Mahmood Bajawa2, Mashhour Hussien Al Qannas2, Polom Karol5, and Delia Cortes Guiral2.

Institution: 1 King Khalid Hosptial, General Surgery, Najran, Saudi Arabia

2 King Khalid Hospital, General Surgery, Najran, Saudi Arabia

3 Hospital La Paz, General Surgery, Madrid, Spain

4 University Hospital of Strasbourg, General Surgery, Strasbourg, France

5 Medical University of Gdansk, Surgical Oncolgy, Gdansk, Poland

### Background

Previous studies have demonstrated that there is remaining disease in the normal-looking peritoneum after a complete CRS in up to 30% of the patients. Fluorescence-guided (FG) surgery is revolutionizing oncologic surgery as it can signal in real time primary or metastatic lesions thanks to indocyanine green (ICG) or targeted dyes.

### Methods

The FLUOZEROC study is a prospective, randomized, multicenter trial designed to study the role of fluorescence guided-surgery for peritoneal metastasis of colorectal origin. Our aim is to know how fluorescence can improve the radicality of the cytoreductive surgery.

### Study Design

Study type: Observational(clinical trial); estimated enrolments: 80 patients (40 conventional CRS, 40 fluorescence-guided CRS: 20 ICG, 20 ICM-137); Intervention: Half of the patients undergoing complete CRS must be evaluated with fluorescence, the aim is to determine the percentage of undetected peritoneal metastasis; After complete FG CRS random biopsies will be taken from the remaining ”normal looking peritoneum”; Patients will be randomized according to KRAS status to be in the standard, the Fluo-ICG or the Fluo-EMI-137 arm. Outcome measures: Primary outcome:Comparison of standard vs fluorescent peritoneal metastases detection; secondary outcome: Several secondary endpoints will be analyzed, including survival.

### Conclusion

Our aim is to demonstrate how fluorescence should turn into an essential tool to accomplish a real CC0.

## OVARIAN CANCER PERITONEAL METASTASES

**Abstract OVCA-01**

**DESCRIPTIVE REVIEW OF CURRENT PRACTICES IN PATIENTS WITH OVARIAN CANCER TREATED BY PIPAC: A MULTICENTRIC RETROSPECTIVE COHORT OF 238 PATIENTS**

Authors: Amaniel Kefleyesus, Aditi Bhatt, Cecilia Escayola, Vladimir Khomyakov, Martin Hübner, Marc Reymond, Rene Thieme, Olivia Sgarbura, Wouter Willaert, Wim Ceelen, Andrea Di Giorgio, Olivier Glehen, Manuela Robella, and Naoual Bakrin

Institution: Lyon University Hospital, Lyon Sud, France

### Background

Ovarian cancer (OC) is the leading cause of death among women diagnosed with gynecological cancer. We reported the current practice of PIPAC in patients with recurrent or progressive OC.

### Methods

International multicentric retrospective study in 18 centers including patients treated palliatively with PIPAC for PM of ovarian origin. All patients were initially treated appropriately outside any clinical trial setting. Clinical practices were analyzed in terms of descriptive feasibility and morbidity of PIPAC.

### Results

Between 2005–2020, 238 patients were included. Mean age was 62.3 years (SD 9.8), 86% ECOGscore was ≤ 1. Platinum sensitivity data could not be analyzed (high missing data rate). While 2/3 had cytoreductive surgery, 6% had HIPEC, before PIPAC. Mean delay from PM diagnosis to PIPAC was 25.9 months (SD 26.5). Forty-one percent received at least 2 PIPAC which was combined with systemic chemotherapy in 49 % of patients. Patients had high tumor load at 1st PIPAC with peritoneal cancer index (PCI) of 20 (SD 10) versus 17 (SD 11) after 2 PIPAC. Morbidity at 30-days was 25% with 7% Clavien grade ≥ 3b and a mortality rate of 1.7%. Median overall survival was 18 months.

### Conclusion

PIPAC is a feasible treatment with low morbidity and mortality. A trend towards PCI improvement seems to be apparent after 2 PIPAC.

## Abstract OVCA-02

**ASSESSING HISTOLOGY STRUCTURES BY EX VIVO MR MICROSCOPY AND EXPLORING THE LINK BETWEEN MRM-DERIVED RADIOMIC FEATURES AND HISTOPATHOLOGY IN OVARIAN CANCER**

Authors: Marion Tardieu1, Yulia Lakhman2 Lakhdar Khellaf3, Maida Cardoso4, Olivia Sgarbura5, Pierre-Emmanuel Colombo5, Mireia Crispin-Ortuzar6,7, Evis Sala6,7, Christophe Goze-Bac4, and Stephanie Nougaret1,8*

Institution: Montpellier Cancer Research institute (IRCM), INSERM U1194, University of Montpellier, Montpellier, France; marion.tardieu@umontpellier.fr 2 Departments of Radiology, Memorial Sloan Kettering Cancer Center, New York, NY, USA 3 Department of Pathology, Montpellier Cancer Institute (ICM), Montpellier, France 4 BNIF facility, L2C, UMR 5221, CNRS, University of Montpellier, Montpellier, France; mai-da.cardoso@umontpellier.fr; christophe.goze@umontpellier.fr 5 Department of Surgical Oncology, Montpellier Cancer Institute (ICM), Montpellier, France 6 Cancer Research UK, Cambridge Institute, University of Cambridge, Cambridge, United Kingdom 7 Department of Radiology, University of Cambridge, Cambridge, United Kingdom. 2 Department of Radiology, Montpellier Cancer Institute (ICM), Montpellier, France

### Background

The value of MR radiomic features at a microscopic scale has not been explored in ovarian cancer. The objective of this study was to probe the associations of MR microscopy (MRM) images and MRM-derived radiomic maps with histopathology in high-grade serous ovarian cancer (HGSOC).

### Methods

Nine peritoneal implants from 9 patients with HGSOC were imaged ex-vivo with MRM using a 9.4 TMR scanner. All MRM images and computed pixel-wise radiomics maps were correlated with the slice-matched stroma and tumor proportion maps derived from whole histopathologic slide images (WHSI) of corresponding peritoneal implants. Auto-mated MRM-derived segmentation maps of tumor and stroma were constructed using holdout test data and validated against the histopathologic gold standard.

### Results

Excellent correlation between MRM images and WHSI was observed (Dice index= 0.77). Entropy, correlation, difference entropy, and sum entropy radiomic features were positively associated with high stromal proportion (r= 0.97,0.88,0.81, and 0.96, respectively, p<0.05). MR signal intensity, energy, homogeneity, auto correlation, difference variance, and sum average were negatively associated with low stromal proportion (r=−0.91,−0.93,−0.94,−0.9,−0.89,−0.89, respectively, p<0.05). Using the automated model, MRM predicted stromal proportion with an accuracy ranging from 61.4% to 71.9%.

### Conclusion

In this hypothesis-generating study, we showed that it is feasible to resolve histologic structures in HGSOC using ex vivo MRM at 9.4 T and radiomics.

## Abstract OVCA-03

**ROLE OF PIPAC IN REFRACTORY MALIGNANT ASCITES IN PATIENTS WITH UNRESECTABLE PERITONEAL METASTASIS**

Authors: Dr. Priya Kapoor, Dr. Somashekhar SP, Dr. Shabber Zaveri, Dr. Ashwin KR, Dr. Rohit Kumar, Dr. Amit Rauthan, and Dr. Sushmita

Institution: Manipal Comprehensive Cancer Center, Bengaluru, India

### Background

Role of PIPAC in refractory malignant ascites in patients with unresectable PM. PIPAC is a new treatment modality with good safety profile and promising early response rates for PM of various origins. The aim of this study was to examine the efficacy of PIPAC in palliation of refractory malignant ascites who were unsuitable for cytoreductive surgery.

### Methods

Between June 2018 to December 2020, 80 patients with unresectable peritoneal metastasis underwent PIPAC. The patient’s demographics, biochemical parameters, peri operative findings, PRGS, adverse events, and outcomes were prospectively recorded.

### Results

Out of the 80 cases, 60% patients had ascites. A total of 37.5% patients had ascitic fluid more than 1000ml. After the 3rd PIPAC 72.9% of patients had resolution of ascites. 62.5% had malignant cells on cytology at first PIPAC while after 3 rd PIPAC 33.33% patients had malignant cells on cytology.

### Conclusion

Our study concludes that PIPAC is a safe and effective method for palliation of refractory ascites in patients with unresectable peritoneal metastasis.

## Abstract OVCA-04

**CORRELATION BETWEEN BIOCHEMICAL PARAMETERS AND PERITONEAL REGRESSION GRADING SCORES ON OUTCOMES IN PATIENTS UNDERGOING PIPAC IN UNRESECTABLE PERITONEAL METASTASIS OF OVARIAN ORIGIN**

Authors: Dr. Priya Kapoor, Dr. Somashekhar SP, Dr. Shabber Zaveri, Dr. Ashwin KR, Dr. Rohit Kumar, Dr. Amit Rauthan, and Dr. Sushmita

Institution: Manipal Comprehensive Cancer Center, Bengaluru, India

### Background

PIPAC is a new treatment modality with good safety profile and promising early response rates for PM of various origins. The aim of this study was to analyze the relationship between biochemical parameters and PRGS on outcomes in patients revieing PIPAC for patients suffering from metastatic ovarian malignancies.

### Methods

Between June 2018 to December 2020, 42 patients with unresectable peritoneal metastasis of ovarian origin underwent pressurized aerosol solution with cisplatin and DXR at 37 C and 12 mmHg for 30 minutes was performed every 6 weeks. The patient’s demographics, biochemical parameters, perioperative findings, PRGS, adverse events, and outcomes were prospectively recorded.

### Results

Out of the 42 cases, 45.23% patients were with PFS and remaining patients had a progressive disease. A total of 69.04% of patients had improvement in PRGS score after 3rd PIPAC. There was no alteration in the serum creatinine and liver enzymes post PIPAC procedure. It was found that there was a statistically significant correlation between CRP, CA – 125, and PRGS groups. Our study shows that decreased CRP, CA – 125, PCI, ascites, and PRGS had a better PFS.

### Conclusion

Our study concludes that radiological response, CRP and CA125 levels significantly correlated to progression free survival in patients with unresectable peritoneal metastases of ovarian origin. Improvement in CRP and CA125 levels significantly correlated with better PRGS scores.

## Abstract OVCA-05

**OUTCOMES OF COMPLEX MULTI-VISCERAL COMPLETE CYTOREDUCTIVE SURGERY AND HYPERTHERMIC INTRAPERITONEAL CHEMOTHERAPY IN ADVANCED RECURRENT GYNECOLOGICAL MALIGNANCIES**

Authors: Dr Sabrina Cheok, Dr Jolene Wong, Dr Johnny Ong, and Dr Claramae Chia

Institution: Singapore General Hospital

### Background

A total of 75% of ovarian cancer patients present with disseminated peritoneal metastases. Optimal cytoreduction is paramount and has significant implications on survival. We aim to evaluate the effect of PCI and multiviscera resection (MVR) in the setting of advanced recurrent ovarian cancer and determine its impact on survival and morbidity outcomes.

### Methods

A retrospective review of recurrent advanced ovarian cancer patients who underwent CRS and HIPEC between 2005–2019 at Singapore General Hospital was performed. Pearson chi-square test and Kaplan–Meier curves were used to investigate the relationship between MVR and morbidity/survival outcomes. MVR is defined as resection of more than 1 organ.

### Results

A total of 53 patients underwent complete CRS and HIPEC (CC0/1). 35 patients had MVR, of whom 85.7% of had diaphragmatic involvement. MVR group had significantly higher rates of major postoperative morbidity compared to the non MVR group (93.8% vs 6.2%, p=0.05) but no differences in survival outcomes was observed (54 vs 53 months, p=0.334).

### Conclusion

High tumor burden involving the upper abdomen is common in advanced recurrent ovarian cancer. These patients often require MVR to achieve optimal cytoreduction. Though patients requiring MVR experience higher morbidity, if complete cytoreduction is achieved, MVR does not result in worse survival outcomes compared to those with lower disease burden.

## Abstract OVCA-06

**CRS+HIPEC IN ADVANCED EPITHELIAL OVARIAN CANCER – AUDIT OF PAN INDIA HIPEC REGISTRY: AN ISPM2 COLLABORATIVE STUDY**

Authors: Dr Somashekhar SP, Dr Rohit Kumar C, Dr SVS Deo, Dr MD Ray, Dr Rupinder Sekhon, Dr. Amita Naithani, Dr Harit Kumar Chaturvedi, Dr Sanjeev Kumar, Dr Subramanyeshwar Rao, Dr Rajagopalan Iyer, Dr Amit Gandhi, Dr Kalpana Kothari, Dr Rama Joshi, Dr Rashmi Rekha Bora, Dr Ganesh, Dr Hemanth, and Dr Ashwin KR.

Institution: Indian Society of Peritoneal Surface Malignancy

### Background

CRS + HIPEC for advanced epithelial ovarian cancer has showed better outcomes. Most of the data is from western world. In resource constrained setting it’s been increasingly adopted since last few years.

### Methods

ISPM2 is a registered society dedicated to advancing the knowledge and practice of management of PM in India. A prospectively maintained HIPEC registry by the society collects and maintains data across India involving 15 centers.

### Results

Out of 1470, 23.3%, 46.6%, and 30 % were upfront, interval and recurrent cancers. Mean age was 54.5±10.74, PCI 12.2±4.57. A total of 80.9% had CC0 resection. 60.31% practiced semi open HIPEC. Median duration of surgery 9.5 hours, blood loss 1100ml, ICU stay 2.8 days, hospital stay 10.54 days. A total of 64.3% had selective parietal peritonectomy, 38.4% diaphragm resection, 57.05 bowel resection rate, 11.1% stoma rate, and 23.6% multivisceral resection rate. Overall G3–G4 morbidity 26.7%, surgical morbidity 21.5%, and anastomotic leak 8.4%. A total of 30 day mortality 3.6%. Median DFS was 26, 33, and 16 months in upfront, interval and recurrent group, respectively. Median OS 50 months in interval group and yet to be achieved in upfront and recurrent group.

### Conclusion

CRS-HIPEC for advanced epithelial ovarian cancer is safe and feasible even in resource constrained setting with improved outcomes and acceptable morbidity. A dedicated team and regular audit with HIPEC registry helps in standardizing the protocol and establishing uniformity across centers.

## Abstract OVCA-07

**PRESSURIZED INTRA-PERITONEAL AEROSOL CHEMOTHERAPY (PIPAC) VS. INTRAVENOUS CHEMOTHERAPY FOR UNRESECTABLE PERITONEAL METASTASES SECONDARY TO PLATINUM RESISTANT OVARIAN CANCER – INTERIM ANALYSIS OF INDIAN RCT**

Authors: Dr Somashekhar SP, Dr Rohit Kumar C, Dr Priya Kapoor, Dr Amit Rauthan, Dr Poonam Patil, Dr Sushmitha Rakshit, Dr Reddi Prasad Yadavalli, Dr Sudarshan Rawat, and Dr Ashwin KR

Institution: Manipal Comphrensive Cancer Center, Manipal Hospital, Bengaluru

### Background

Platinum resistant ovarian cancer has dismal prognosis with the present standard of care in form of IV chemotherapy. PIPAC has shown improved response rate and quality of life in these patients.

### Methods

The trial is registered with Clinical Trials Registry – India (CTRI) REF/2018/08/021223. Interim analysis is presented here. Primary endpoint was to assess the objective response rate (With RECIST 1.1) between PIPAC and IV chemotherapy arm. Secondary endpoints were to assess quality of life (QLQ C-30) and morbidity (CTCAE 4.0 and Clavien Dindo).

### Results

A total of 40 patients underwent 105 PIPAC applications with nearly 25 (62.5%) had 3 cycles completed. A total of 40 patients underwent IV chemotherapy same time with nearly 23 (57.5%) having received at least 5 cycles. Mean age was 57.3±8.05, PCI 24.45±6.39, with nearly 72.5% of patients having received atleast 2 lines of prior chemotherapy and 60 % having ascites. The objective response rate is 66.6% versus 22.5%, grade 3-4 events were 10.0% vs. 35.7 % in PIPAC, and IV arm, respectively. Histological regression was seen in 67.5% of patients with 3 cycles PIPAC. Functional, symptom, and global health score at day 120 was significantly better with PIPAC arm when compared to IV arm.

### Conclusion

PIPAC is safe and feasible for patients with unresectable platinum resistant ovarian cancer. PIPAC showed better objective response rates when compared to chemotherapy arm with low morbidity and improved quality of life.

## Abstract OVCA-08

**PRIMARY CYTOREDUCTIVE SURGERY (CRS) COMBINED WITH SIMULTANEOUS PRESSURIZED INTRAPERITONEAL AEROSOL CHEMOTHERAPY (PIPAC) FOLLOWED BY SYSTEMIC CHEMOTHERAPY AND PIPAC SESSIONS FOR STAGE III EPITHELIAL OVARIAN CANCER: PRELIMINARY RESULTS FROM PHASE-2 STUDY**

Authors: Aleksey Dzasokhov 1 and Vladimir Khomyakov 2

Institution: 1-Moscow Regional Clinical Oncological Dispensary, Balashikha, Russia 2- P.A. Hertsen Moscow Research Oncological Institute – branch of the National Medical Research Center of Radiology, Moscow, Russia

### Background

Patients with synchronous PM of gynecological origin have a high recurrence rate and poor prognosis. CRS combined with PIPAC is a novel approach with a goal of improving recurrence-free survival in ovarian cancer.

### Methods

Treatment protocol includes suboptimal primary CRS combined with simultaneous PIPAC. After finishing open procedure two 10 mm trocars were placed in the right and left flunk and abdomen incision was closured. A 12 mm Hg CO_2_ pneumoperitoneum was established. The solution of cisplatin 10.5 mg/m2 and DXR 2.1 mg/m2 was aerosolized to the abdomen cavity. The exposure was 30 minutes. Adjuvant chemotherapy (TC) started on the second week after surgery. Systemic treatment was alternated with two laparoscopic PIPAC sessions after 2-nd and 4-th systemic courses.

### Results

A total of 13 patients received primary CRS with PIPAC. There were no postoperative deaths and no major complications (Grade ≥3). Among them 12 patients had 2 PIPAC and 6 patients had all 3 PIPAC sessions. Initial PCI ranged from 12 to 39. On the second PIPAC PCI was 0 in all 12 patients. Multiple biopsies showed complete response in peritoneal lesions in all 6 patients on PIPAC 3.

### Conclusion

PIPAC combined with CRS and standard systemic CT in primary ovarian cancer is safe, easy to perform and well tolerated. Our first experience showed promising results. Further studies are required to confirm the safety and efficacy of the novel approach.

## MESOTHELIOMA

**Invited lecture**

**IMAGE-BASED ASSESSMENT OF MESOTHELIOMA TUMOR BURDEN AND RESPONSE TO THERAPY**

Authors: Samuel G. Armato III

Institution: The University of Chicago, IL, USA

The dominant imaging modalities for mesothelioma assessment are CT, MRI, and positron emission tomography (PET). CT is the most common imaging examination, providing structural information about mesothelioma tumor and adjacent anatomic structures; functional information regarding tumor perfusion also may be captured through dynamic, contrast-enhanced CT, which has recently been extended to mesothelioma in a research setting as a potential marker for tumor response. MRI inherently captures both structural and functional information, with a wide variety of pulse sequences available to cover a broad range of applications, including the potential for discrimination between epithelioid and sarcomatoid histologic subtypes in mesothelioma using diffusion-weighted MRI. PET imaging captures functional information through such mesothelioma-relevant mechanim2s as metabolim, cell proliferation, and tumor hypoxia; total glycolytic volume specifically has proven beneficial in mesothelioma. The current clinical standard for mesothelioma tumor measurement and response assessment is the modified RECISTs (mRECISTs) guidelines, which are tailored to the unique morphology of mesothelioma; inconsistent interpretation and misinterpretation of the modified measurement approach led to the more recent publication of mRECIST 1.1. Tumor volume may eventually replace the linear measurements of mRECIST.

## Abstract MESO-01

**PRESSURIZED INTRAPERITONEAL AEROSOL CHEMOTHERAPY (PIPAC) FOR DIFFUSE MALIGNANT PERITONEALM MESOTHELIOMA IS ASSOCIATED WITH PROMISING ONCOLOGICAL OUTCOMES**

Authors: Olivia Sgarbura1,2, Alix Bouillin1, Jean-Baptiste Delhorme3, Tarkan Jäger4, Océane Massol5, Marc Pocard6, Vahan Kepenekian7, and the ISSPP PIPAC cohort study group

Institution: 1 Department of Surgical Oncology, Cancer Institute of Montpellier, University of Montpellier, Montpellier, France 2 IRCM, Institut de Recherche en Cancérologie de Montpellier,

INSERM U1194, Université de Montpellier, Institut régional du Cancer de Montpellier, Montpellier, F-34298, France. 3 Department of Surgery, CHU Strasbourg, Strasbourg, France 4 Department of Surgery, Paracelsus Medical University Salzburg 5 Department of Biometrics, Cancer Institute of Montpellier, University of Montpellier, Montpellier, France 6 Department of Surgery, CHU Pitié Salpêtriere, Paris, France 7 Department of Surgery, CHU Lyon Sud, Lyon, France

### Background

PIPAC is a novel IP drug delivery method, implemented for PM of various origins. We aimed to analyze oncological outcomes after PIPAC for unresectable diffuse malignant peritoneal mesothelioma (DMPM).

### Methods

This retrospective cohort study included consecutive patients with DMPM treated in experienced PIPAC centers (>60 procedures) worldwide. The primary outcome measure was OS. Secondary outcome measures included progression free survival (PFS) and response to treatment through radiological (RECIST criteria), visual (peritoneal cancer index -PCI), histological (cytology and peritoneal regression grading system -PRGS), and clinical variables.

### Results

Final analysis included 80 consecutive patients (240 PIPAC procedures) from 15 centers. For 67% of the patients, PIPAC was applied in the first line. Median OS was 35 months (CI 19–118) from time of diagnosis and 22 months (CI 14–49) from the start of PIPAC. Median PFS was 19 months (CI 15–35) from time of diagnosis and 13 months (CI 8–16) from the 1st PIPAC. A total of 42 patients (52.5%) had ≥3 procedures. Response to treatment at PIPAC3 was as follows: RECIST: partial response 22.5%, stability 55%; median PCI: 23 vs. 29 (p=NS); mean PRGS: 2.3±0.7; positive cytology 51% vs 74% (p=0.02).

### Conclusion

Patients with DMPM showed objective response to PIPAC treatment and promising oncological outcomes.

## Abstract MESO-02

**MULTICYSTIC PERITONEAL MESOTHELIOMA TREATED WITH CYTOREDUCTIVE SURGERY FOLLOWED OR NOT BY HYPERTHERMIC INTRAPERITONEAL CHEMOTHERAPY: RESULTS FROM A LARGE MULTICENTRIC COHORT**

Authors: KEPENEKIAN Vahan, PERON Julien, GOERE Diane, SGARBURA Olivia, DELHORME Jean- Baptiste; EVENO Clarisse, BENZERDJEB Nazim, BONNEFOY Isabelle, VILLENEUVE Laurent, ROUSSET Pascal, ABBOUD Karine, POCARD Marc, and GLEHEN Olivier

Institution: Hospices civils de Lyon

### Background

MCPM is a rare, slowly growing, condition prone to recur after surgery. The role of HIPEC added to complete CRS remains controversial and difficult to assess. As patients are mostly reproductive age women, surgical approach, and fertility considerations are important aspects of the management.

### Methods

MCPM patients treated with CRS over a 1999–2019 period were included. A special focus on HIPEC, mini-invasive approach and fertility considerations was performed.

### Results

Overall 60 patients were included with a median PCI of 10 (4-14) allowing 97% of complete surgery, followed by HIPEC in 82% of patients. A quarter of patients had a laparoscopic approach. Twelve patients (20%) recurred with a 3-year recurrence free survival of 84.2% (95% confidence interval 74.7 to 95.0). The hazard of recurrence was numerically reduced among patients receiving HIPEC however not statistically significant (hazard ratio 0.41, 0.12 to 1.42, p=0.200). A severe postoperative adverse event occurred in 22% of patients with 5 patients submitted to a subsequent reoperation. Among 4 patients with a childbearing desire, 3 were successful.

### Conclusion

MCPM patients treatment should aim at a complete CRS. The intraoperative treatment options as laparoscopic approach, fertility function sparing and HIPEC should be discussed in expert centers to propose the most appropriate strategy.

## Abstract MESO-03

**NON RESECTABLE MALIGNANT PERITONEAL MESOTHELIOMA TREATED WITH PRESSURIZED INTRAPERITONEAL AEROSOL CHEMOTHERAPY COMBINED WITH SYSTEMIC CHEMOTHERAPY COULD LEAD TO SECONDARY COMPLETE CYTOREDUCTIVE SURGERY**

Authors: KEPENEKIAN Vahan, PERON Julien, BONNEFOY Isabelle, ALYAMI Mohammad, VILLENEUVE Laurent, BAKRIN Naoual, ROUSSET Pascal, YOU Benoit, BENZERDJEB Nazim, and GLEHEN Olivier

Institution: Hospices Civils de Lyon

### Background

DMPM is an aggressive peritoneal disease. The association of CRS and HIPEC is recommended in selected patients providing the best long-term outcomes. PIPAC combined to systemic chemotherapy (sCT) has been recently proposed as neo-adjuvant strategy.

### Methods

Consecutive primary or recurrent nonresectable DMPM patients who received ≥1 PIPAC in alternation with sCT were included.

### Results

Overall, 26 DMPM patients were treated including 20 of them with no previous CRS. Most patients (85%) had symptoms, comprising 9 with perceptible ascite. A total of 79 PIPAC procedures were performed, half of patients receiving ≥3 PIPAC. Ten adverse events (AEs) were reported among 8 patients (31%), including 2 severe AEs corresponding to digestive perforations. The initial symptoms were improved in 32% of these patients whilst a control of ascites was noted in 46%. Fourteen patients (54%) were secondarily treated by CRS-HIPEC with one being considered as complete resection. After a median follow-up of 29.6 months, the median OS was 12 months. The median PFS was significantly better among resected patients compared to those not resected 33.5 months vs. 7.4 months (HR 0.18, 95% CI 0.06-0.755, p<0.001).

### Conclusion

PIPAC is feasible in a neo-adjuvant intent for initially nonresectable DMPM patients, and could facilitate complete secondary resection.

## Abstract MESO-04

**IN VITRO BREATH ANALYSIS OF MALIGNANT PLEURAL MESOTHELIOMA CELL LINES**

Authors: Liam David Little (1), Vikki Amanda Carolan (1), Kathryn Elizabeth Allen (1), Laura Margaret Cole (1), Martina Tripari (2), Jenna Kenyani (2), Joe Sacco (3), Judy Coulson (2), and Sarah Haywood-M2all (1)

Institution: 1: Biomolecular Sciences Research Center, Sheffield Hallam University, Sheffield, UK.

2: Molecular Physiology and Cell Signalling, Institute of Systems Molecular & Integrative Biology, University of Liverpool, Liverpool, UK. 3: Molecular and Clinical Cancer Medicine, Institute of Systems Molecular & Integrative Biology, University of Liverpool, Liverpool, UK.

### Background

Malignant pleural mesothelioma (MPM) is an aggressive pleural cancer with a challenging diagnosis that precludes curative treatment. Volatile organic compounds (VOCs) in exhaled breath are believed to improve MPM detection.

### Methods

We have used solid-phase microextraction (SPME) and gas chromatography-mass spectrometry (GCMS) to identify VOCs from MPM and nonmalignant control cell lines. This research is further developed to identify VOCs released from a non-malignant mesothelial cell line, MET-5A modified to have stably reduced BAP1 expression (BAP1w-/KO). BAP1 is the most commonly mutated gene in MPM; this investigation aims to identify VOCs associated with BAP1 status as potential early diagnostic biomarkers. VOCs were sampled from MET5A BAP1w- /KO headspace using SPME and GCMS. VOCs were compared to previous MPM cell line results to identify changes associated with BAP1.

### Results

Previously, our multivariate statistical analysis results (PCA and OPLS) showed separation between epithelioid (NCI-H28) and biphasic (MSTO-211H) MPM cells and MET5A controls. VOCs were identified from MET5A BAP1w-/KO cells with further analysis required to confirm compounds associated with BAP1 mutation.

### Conclusion

Breath analysis of VOCs has the potential to dramatically improve MPM diagnosis, providing a reliable and noninvasive alternate to current methods. The *in vitro* headspace model is an important tool in VOC analysis, detecting VOCs associated with BAP1 mutations identifies potential biomarkers for MPM diagnosis.

## PSEUDOMYXOMA PERITONEI

**Invited lecture**

**NEW PMP PATHOLOGICAL GRADING AND STAGING**

Prof. Norman John Carr, MD PhD

Institution: Peritoneal Malignancy Institute, Basingstoke, UK

Pseudomyxoma peritonei (PMP) is now classified in 4 diagnostic groups:

1. Acellular mucin (AM). In the context of appendiceal mucinous neoplasia the risk of progressive disease is very low if the ip mucin is acellular. The 10-year survival is about 90%. AM is ungraded in the WHO classification. Note that other mucinous lesions, such as ovarian cystadenomas, can also produce AM.
2. Low grade PMP (low grade mucinous carcinoma peritonei). This is characterized by minimal cytological atypia, low cellularity and rare mitotic figures. The WHO grade is G1. The usual primary is a low grade appendiceal mucinous neoplam2.
3. High grade PMP (high grade mucinous carcinoma peritonei). This has increased atypia and tends to be more cellular. It is generally categorized G2, although very rare examples with sheets of poorly differentiated cells can be classified G3.
4. High grade PMP with signet ring cells (high grade mucinous carcinoma peritonei with signet ring cells). At least 10% of cells should show signet ring morphology for this classification because of interobserver variation in identifying them when they are scanty. It has the worst prognosis and is G3.

In TNM8, PMP of appendiceal origin is classified as:

pM1a if only acellular mucin is found.
pM1b if ip mucin contains neoplastic epithelial cells; this encompasses ‘typical’ PMP including ovarian involvement.
pM1c if there are nonperitoneal metastases, implying hematogenous or distant lymphatic spread.

## Abstract PMP-01

**M2ALL BOWEL INVOLVEMENT TOPOGRAPHY AND SUCCESS OF CYTOREDUCTIVE SURGERY WITH HYPERTHERMIC INTRAPERITONEAL CHEMOTHERAPY IN PATIENTS WITH PERITONEAL SURFACE MALIGNANCIES**

Authors: Andrei Nikiforchin, MD;, Armando Sardi, MD, FACS; Michelle Sittig, RN; Ekaterina Baron, MD; Felipe Lopez-Ramirez, MD; Carol Nieroda, MD; and Vadim Gushchin, MD, FACS

Institution: The Institute for Cancer Care, Mercy Medical Center, Baltimore, MD, USA.

### Background

The success of CRS/HIPEC for peritoneal surface malignancies (PM2) is determined by complete cytoreduction (CC), which is often limited by small bowel (SB) involvement. We assessed the impact of SB involvement patterns on CRS/HIPEC failure.

### Methods

We conducted a case-control study using a single-center database (2018-2020). PM2 patients with involved SB undergoing a CRS/HIPEC attempt were assigned to palliative/aborted (“cases”) and successful CC-0/1/2 (“controls”) procedure groups. SB disease patterns included lesions on bowel serosa, mesenteric border, and mesentery. Logistic regression evaluated the impact of patterns on failed CRS/HIPEC attempt.

### Results

Overall 131 patients were identified: 19 cases, 112 controls. Groups were balanced by age, sex, and primaries (appendiceal, colorectal, ovarian cancer). Median PCI was higher in “cases”: 39 (IQR: 35–39) vs 27 (IQR: 17–33), p<0.001. SB patterns by “cases” vs “controls” were: serosal 84 vs 73% (p=0.40), mesenteric border 95 vs 59% (p=0.003), mesentery 100 vs 97% (p=1.00). Neither morbidity (p=0.69) nor mortality (p=0.38) was different. Mesenteric border involvement (OR 12.5; 95%CI: 1.5–104.3) was associated with failed CRS/HIPEC in adjustment to other factors.

### Conclusion

Mesenteric border lesions are associated with failed CRS/HIPEC attempt for PM2 with SB involved whereas serosal and mesenteric lesions do not limit the achievement of CC.

## Abstract PMP-02

**HYPERTHERMIC INTRATHORACIC CHEMOTHERAPY COMBINED TO ITERATIVE CYTOREDUCTIVE SURGERY TO TREAT A PLEURAL INVOLVEMENT FROM PSUDOMIXOMA PERITONEI**

Authors: Filippo Lococo, MD1, Andrea Di Giorgio, MD2, Amedeo Iaffaldano, MD1, Giovanni Schinzari, MD3, Diomira Tabacco, MD1, Jessica Evangelista, MD1, Paola Aceto, MD4, Carlo Abatini, MD2, Liliana Sollazzi, MD4, and Stefano Margaritora, MD1

Institution: 1 Unit of Thoracic Surgery, 2 General Surgery, 3 Oncology Unit, 4 Anaesthesiology Unit, Fondazione Policlinico Universitario "A. Gemelli",IRCCS, Università Cattolica del Sacro Cuore, Rome, Italy

### Background

Pseudomyxoma peritonei (PMP) is an uncommon disease with locally-invasive attitude. Intrathoracic spread is rarely reported and its management extremely challenging.

### Case report

A 51-year-old caucasian female presented with left pleural carcinosis 9-months after two sequential abdominal surgical procedures combined with HIPEC for low-grade PMP. Cytoreductive surgery (pleurectomy/decortication) was followed by 60-minutes hyperthermic intrathoracic chemotherapy mitomycin-C (215mg/m2) infusing at same temperature (42°C) and intrapleural pression (2-4 mmH20). No intraoperative complication occurred, the post-op stay was uneventful and no sign of recurrence was observed 9-months after surgery.

### Conclusion

Cytoreductive thoracic surgery and hyperthermic chemotherapy (HITHOC) could be a feasible therapeutic option in selected cases.

## Abstract PMP-03

**THE FIRST PROTEIN PROFILE OF PSEUDOMYXOMA PERITONEI: A MOLECULAR CHARACTERIZATION OF MUCIN AND SOLID TUMOR**

Authors: Romero-Ruiz Antonio 1, 2*, Granados-Rodríguez M 1*, Valenzuela Molina Francisca 1, 2; Rufián Andújar Blanca 1, 2; Morón-Márquez Laura 1, Durán Martínez Manuel 1, 2; Rufián Peña Sebastián 1, 2; Casado Adam Ángela 1, 2; Sánchez Hidalgo Juan M 1, 2; Rodríguez Ortiz Lidia 1, 2; Martínez Ana 3, Ortega Salas Rosa 3; and Arjona Sánchez Álvaro 1, 2.

* A RR and M-GR, contributed equally to this job and should be considered as first authors.

Institution: 1 Maimonides Biomedical Research Institute of Córdoba (IMIBIC). Spain 2 General Surgery Unit - Reina Sofia University Hospital. Spain, 3 Pathology Unit, Reina Sofia University Hospital, Spain

### Background

PMP is a rare malignant growth characterized by the progressive accumulation of mucus-secreting tumor cells within the abdomen and pelvis. In the last few years, reference centers have published a survival benefit after a macroscopic cytoreduction following by HIPEC. Despite positive results, a considerable number of patients experience recurrence with a fatal end. In addition, the only available option in case of recurrence is the use of secondary surgeries or the chemotherapy protocols applied in colorectal cancer, which has not shown positive evidence in PMP. In this context, molecular characterization is mandatory, not only to classify the tumor but also to develop personalized therapeutic strategies. However, the main obstacle reaching this milestone is the mucin, composed of a high concentration of glycoproteins, mainly MUC-2. To our knowledge, no study has been published about the protein profile of PMP, neither mucin nor solid tumor.

### Methods

We have developed a new protocol based on a HPLC system followed by a mass spectrometry platform.

### Results

This strategy has enabled us to get a library with more than 1000 protein species from mucin and more than 1500 from the solid tumor; moreover, our preliminary differential protein expression analyses are reporting new knowledge about the pathophysiology of this malignant disease.

Source of Funding: National Institute of Health Carlos III. Reference: PI19/01603.

## Abstract PMP-04

**INTRATUMORAL BROMELAIN AND ACETYLCYSTEINE FOR RECURRENT AND UNRESECTABLE PSEUDOMYXOMA PERITONEII. A PHASE I/II, UNICENTRIC STUDY**

Authors: Lidia Rodríguez Ortiz, Antonio Romero Ruiz, Juan Manuel Sánchez, Ángela Casado Adam, Sebastián Rufián Peña, and Álvaro Arjona Sánchez

Institution: Universitary Hospital Reina Sofía, Cordoba, Sapin

### Background

Mucinous neoplams with peritoneal spread constitute an infrequent entity known as pseudomyxoma peritonei. The massive intraabdominal affectation contrast with the optimisticprognosis owing to its histological low grade. Cytoreductive surgery and HIPEC are the standard treatment. Locoregional recurrence occurs in 20–30% of the cases. The scarce success of intravenous chemotherapy compel to iterative surgeries as the best treatment for recurrences.Hence, patients with several surgeries have an inadmissible morbidity risk for another cytoreductionin case of new relapses, being the supportive care the only available treatment. This clinical trial is focused on the treatment for PMP abdominal recurrences in inoperable patients.

The objective is to assess the effectiveness of the combination of Bromelain and Acetylcysteine (BromAc). Its synergistic activity Results in dissolution of tumor-produced mucin both *in vitro* and invivo, along with a cytotoxic effect and improved chemosensitivity.

### Methods

For this phase I/II, unicentric trial, 10 patients will be selected (inoperable abdominal mucin massesor mucin ascites). The BromAc will be administrated as a minimally invasive treatment through apercutaneous catheter directly to the mucin for a total of three days. Doses will be adjusted to the tumor volume.

### Results

We aim to assess the safety, feasibility and effectiveness of the BromAc to dissolve mucin masses, to relieve symptoms and to control tumor progress.

## Abstract PMP-05

**A PROPOSAL FOR MODIFICATION OF PSOGI CLASSIFICATION ACCORDING TO KI-67 PROLIFERATION INDEX IN PSEUDOMYXOMA PERITONEI**

Authors: Álvaro Arjona-Sánchez, Ana Martínez-López Francisca Valenzuela-Molina, Blanca Rufián- Andújar, Sebastián Rufián-Peña, Ángela Casado-Adam, Juan Manuel Sánchez-Hidalgo,Lidia Rodríguez-Ortiz, Francisco Javier Medina-Fernández, Cesar Díaz-López, Melissa Granados- Rodríguez, Rosa Ortega-Salas, Justo P. Castaño, Manuel Tena-Sempere Javier Briceño-Delgado, and Antonio Romero-Ruíz

Institution: Hospital University Reina Sofia, Cordoba, Spain

### Background

PMP is a rare malignancy, which, as classified by PSOGI, remains highly heterogeneous within the high-grade (HG) category when responding to treatment. Molecular profiling of PMP cases might offer improved patient categorization and predict treatment responses.

### Methods

We studied the Ki67 proliferation index and the overexpression of P53 in tissue samples from our historical cohort of HG-PMP. We established, as cut-off, 3rd quartile of each marker toper form univariate and multivariate Cox regression survival analyses.The HG-PMP category was divided in two sub-categories and a new survival analysis was performed.

### Results

A total of 90/117 patients with PMP undergoing CRS and HIPEC were selected for secondary analysis. The survival analysis of the HG-PMP category for preoperative variables showed that Ki67>15% is a negative prognostic factor with a hazard ratio (HR) of 3.20 (1.24–8.25 CI 95%).Accordingly, the HG-PMP group was divided using the Ki67 15% cut-off. The new PSOGI-Ki67variable served as independent prognostic factor for overall survival, HR:3.74 (1.88–7.47 CI95%) and disease free survival, HR: 4.184 (1.79–9.75 CI95%f). The 5-year OS was 100%, 70%, and 24% (p=0.0001), 5-year DFS was 90%, 44%, and 0% for LG-PMP, HG-PMP≤15%, and HG-PMP>15% (p=0.0001).

### Conclusion

Division of the HG-PMP category of the PSOGI classification, according to the Ki67proliferation index, provides two well-defined subcategories, with significant differences in terms of OS and DFS.

## Abstract PMP-06

**PEPTIDE VACCINE TARGETING MUTATED GNAS – A POTENTIAL NOVEL TREATMENT FOR PSEUDOMYXOMA PERITONEI**

Authors: Else Marit Inderberg, Annette Torgunrud, Karianne Giller Fleten, Ben Davidson, Hedvig Vidarsdotter Juul,Nadia Mensali, Christin Lund-Andersen, and Kjersti Flatmark

Institution: Oslo University Hospital, Oslo, Norway

### Background

PMP is a rare, slow-growing abdominal cancer with no efficacious treatment options in nonresectable and recurrent cases. Otherwise rare activating mutations in the GNAS oncogene are remarkably frequent in PMP. The mutated gene product, guanine nucleotide-binding protein α subunit (Gsα), is a tumor neoantigen and potential target for a therapeutic cancer vaccine.

### Methods

Tumor and blood samples were collected from 25 PMP patients undergoing surgery (NCT02073500). GNAS mutations were detected by next-generation targeted sequencing or digital droplet PCR. Immune responses against Gsα mutated (point mutations R201H and R201C) long peptides were tested in blood samples and tumor samples were analyzed for immune cell infiltration by mass cytometry (CyTOF).

### Results

GNAS mutations were found in 22/25 tumors. Strong T cell responses against Gsα mutated peptides were observed in 18/24 PMP patients. CD4 and CD8 T cell infiltration was seen in most tumor samples. A large proportion of T cells expressed immune checkpoint molecules, in particular PD-1 and TIGIT, indicating that these T cells were antigen-experienced.

### Conclusion

Our findings demonstrate preexisting immunity in PMP patients against mutated Gsα which has been insufficient to control tumor growth, possibly due to immune checkpoint upregulation. This provides rationale for exploring Gsα peptide vaccination combined with immune checkpoint inhibition as a possible curative treatment for PMP and other GNAS mutated cancers.

## PANCREATIC AND BILIARY TRACT CANCER PERITONEAL METASTASES

**Abstract HBP-01**

**COMBINED NABPACLITAXEL PRESSURIZED INTRAPERITONEAL AEROSOL CHEMOTHERAPY WITH SYSTEMIC NAB-PACLITAXEL-GEMCITABINE CHEMOTHERAPY FOR PANCREATIC CANCER PERITONEAL METASTASES. A SINGLE-ARM, OPEN-LABEL, PHASE II TRIAL. NAB-PIPAC TRIAL**

Authors: Andrea Di Giorgio, Federica Ferracci, Carlo Alberto Schena, Cinzia Bagalà, Fabio Pacelli, and Stefano Rotolo

Institution: Fondazione Policlinico Universitario Agostino Gemelli IRCCS

### Background

Recently, a dose-escalation study identified the safe ip dose of Nabpaclitaxel (Nab-PTX) for PIPAC a novel method of ip delivery of anticancer drugs. Nab-PTX and gemcitabine (GEM) represent the first-line treatment of metastatic pancreatic carcinoma PM.

### Methods

We designed a phase II open-label study to evaluate the antitumoral activity of NabPTX-PIPAC (Nab- PIPAC) combined with endovenous GEM-NabPTX in terms of pathological tumor response for PM patients affected by PM. The secondary outcomes include safety, radiological tumor response, time-to-progression and overall survival, QoL, nutritional status, and pharmacokinetics of Nab-PIPAC. Patients are scheduled for 3 courses of combined treatment, each consisting of II cycles of endovenous GEM-NabPTX chemotherapy and 1 Nab-PIPAC. Hence, each patient will receive a total of VI cycles of systemic chemotherapy and 3 PIPAC administrations. GEM-NabPTX is administered according to the standard doses for metastatic PM, while ip Nab-PTX is given at the dose of 112.5 mg/m2. Simon’s two-stage design is used for sample size calculation with 11 patients enrolled in the first stage and 5 in the second one (power 80%, alpha 0.2).

### Conclusion

The present trial will clarify if Nab-PIPAC combined with first-line systemic GEM-NabPTX chemotherapy has an antitumoral activity and it is safe for PM patients with PM.

## Abstract HBP-02

**PRESSURIZED INTRAPERITONEAL AEROSOL CHEMOTHERAPY (PIPAC) FOR ISOLATED PERITONEAL METASTASIS OF HEPATOBILIARY-PANCREATIC ORIGIN: A MULTICENTRIC COHORT STUDY ON 101 PATIENTS**

Authors: Giorgi Nadiradze, Wim Ceelen, Andrea di Giorgio, David Orry, Rene Thieme, Martin Hübner, Olivier Glehen, Olivia Sgarbura, Juanjo Torrent, Manuela Robella, Marc A. Reymond, and ISSPP PIPAC study group

Institution: PIPAC Study group, International Society for the Study of Pleura and Peitoneum

### Background

Isolated PM of pancreatic (PANC) and hepatobiliary (HB) origin are rare and perceived as lethal.

### Methods

This is a multicentric retrospective study with anonymized consecutive cases. Imputation for missing values using k-nearest neighbors.

### Results

A total of 101 patients with isolated PM were treated, with comparable characteristics between PANC (n=60) and HB (n=41) patients. A total of 45 patients had synchronous, 56 metachronous PM. A total of 47 patients were in the 1st, 28 in the 2nd, and 18 in the 3rd line situation; 8 received no chemotherapy. In total, 240 PIPAC cycles (cisplatin/DXR: 64, OXA: 35, other:2) were administered, 93% patients received chemotherapy in addition to PIPAC. Two access injuries, three complications ≥ 3 Clavien–Dindo, and one hospital death were reported. Median survival from diagnosis was: PANC 27.2 (CI 5-95%: 18.8-35.6) months, and HB 37.6 (17.7-57.5) months (log-rank, p=0.01). Median survival from first PIPAC was: PANC 6.0 (3.8-8.4) months and HB 13.0 (11.1-14.9) months (p=0.001). Independent prognostic factors for longer survival were: origin (PANC vs. HB, HR 0.54, p=.02); high number of PIPAC cycles (HR 0.69, p<0.001), high BMI (HR 0.85, p<.001); high PCI (HR 1.09, p<.001), poor ECOG (HR 0.69, p=.02); high number of chemotherapy cycles (HR 0.96, p=.02).

### Conclusion

PIPAC is feasible and safe for isolated PM of PANC and HB origin. Survival after systemic chemotherapy and PIPAC is promising and better for HB compared to PANC.

## Abstract HBP-03

**INTRAPERITONEAL PACLITAXEL COMBINED WITH GEMCITABINE PLUS NAB-PACLITAXEL IN PANCREATIC CANCER PATIENTS WITH PERITONEAL METASTASIS**

Authors: Naminatsu Takahara, M.D., PhD 1), Yousuke Nakai, M.D., PhD 2), Hironori Ishigami, M.D., PhD 3), Kei Saito, M.D., PhD 1), Kazunaga Ishigaki, M.D., PhD 3), Ryunosuke Hakuta, M.D., PhD 2), Tomotaka Saito, M.D., PhD 1), Tsuyoshi Hamada, M.D., PhD 1), Suguru Mizuno, M.D., PhD 1), Hirofumi Kogure, M.D., PhD 1), and Mitsuhiro Fujishiro, M.D., PhD 1).

Institution: 1) Department of Gastroenterology, Graduate School of Medicine, The University of Tokyo, Tokyo, Japan 2) Department of Endoscopy and Endoscopic Surgery, Graduate School of Medicine, The University of Tokyo, Tokyo, Japan 3) Department of Chemotherapy, The University of Tokyo, Tokyo, Japan.

### Background

Since no survival improvement has been achieved over time in patients with PCPM, a novel treatment option is urgently needed.

### Methods

This is a phase I study of ip PTX combined with GnP to determine the maximum tolerated dose (MTD) and the recommended dose (RD) in patients with chemotherapy-naïve PCPM. Based on the 3 + 3 dose-escalation model, ip PTX, GEM and nab-PTX were administered at doses of 20 or 30 mg/m2, 800 or 1000 mg/m2 and 100 or 125 mg/m2 (level 1, 2, and 3, respectively) on days 1, 8 and 15 in 4-week cycles. Dose-limiting toxicity (DLT) defined as severe adverse events were evaluated during the first cycle of the treatment. Safety and preliminary efficacy were also investigated.

### Results

In total, 12 patients were enrolled. While 2 of the first 6 patients enrolled at level 1 experienced DLTs, no DLT was observed in the next 6 patients enrolled at level 2 and 3. Therefore, RD was determined to be level 3 (ip PTX of 30 mg/m2, GEM of 1000 mg/m2, and nab-PTX of 125 mg/m2). The major grade 3/4 adverse events included neutropenia (58%), anemia (33%), and ip port dysfunction (25%). The response rate was 25% and the median PFS was 5.4 months. The cytological status in peritoneal lavage turned negative in 8 patients (67%).

### Conclusion

Considering the poor outcomes in patients with PCPM, Ip PTX combined with GnP was feasible and potentially effective as a first-line treatment deserves a further evaluation.

